# Nucleolipid Acid-Based Nanocarriers Restore Neuronal Lysosomal Acidification Defects

**DOI:** 10.3389/fchem.2021.736554

**Published:** 2021-08-20

**Authors:** Mathias Brouillard, Philippe Barthélémy, Benjamin Dehay, Sylvie Crauste-Manciet, Valérie Desvergnes

**Affiliations:** ^1^ University of Bordeaux, INSERM U1212, UMR CNRS 5320, Bordeaux, France; ^2^ University of Bordeaux, CNRS, IMN, UMR 5293, Bordeaux, France; ^3^ University Hospital, Bordeaux, France

**Keywords:** nucleolipid, nanocarrier, oil-in-water nanoemulsion, lysosome, pH, acidification, neurodegenerative disease

## Abstract

Increasing evidence suggests that lysosomal dysfunction has a pathogenic role in neurodegenerative diseases. In particular, an increase in lysosomal pH has been reported in different cellular models of Parkinson’s disease. Thus, targeting lysosomes has emerged as a promising approach. More specifically, regulating its pH could play a central role against the neurodegeneration process. To date, only a few agents specifically targeting lysosomal pH are reported in the literature, partly due to the challenge of crossing the Blood-Brain-Barrier (BBB), preventing drug penetration into the central nervous system (CNS). To develop chronic treatments for neurodegenerative diseases, crossing the BBB is crucial. We report herein the conception and synthesis of an innovative DNA derivative-based nanocarrier. Nucleolipids, carrying a biocompatible organic acid as an active ingredient, were designed and synthesized as prodrugs. They were successfully incorporated into an oil-in-water nanoemulsion vehicle to cross biological membranes and then release effectively biocompatible acidic components to restore the functional lysosomal pH of neuronal cells. Biological assays on a genetic cell model of Parkinson’s disease highlighted the non-toxicity of such nucleolipids after cellular uptake and their ability (at c = 40 µM) to fully restore lysosomal acidity.

## Introduction

Lysosomes play a crucial role in many cellular processes. These membrane-bound acidic organelles present in the cytosol of all eukaryotic cells are particularly implicated in the autophagy-lysosomal pathway (ALP) (Luzio et al., 2007), (Yim and Mizushima, 2020). The ALP is the main mechanism for the degradation of intracellular material and, notably, long-lived proteins and old or damaged organelles. The degradation process requests a crucial acidic lysosomal pH (4.5–5.5), which optimizes the concerted action of more than 60 hydrolases (Settembre et al., 2013) and ensures fusion between lysosomes and autophagosomes (Kawai et al., 2007). The significant role of the lysosome pH has become evident in many pathologies such as lysosomal storage disorders (Marques and Saftig, 2019), immunological diseases, diabetes, cancer, and neurodegenerative diseases (Koh et al., 2019), (Colacurcio and Nixon, 2016), making the lysosome a promising therapeutic target (Hafner Česen et al., 2012), (Bonam et al., 2019), (Peng et al., 2019). In the specific case of neurodegenerative diseases, particularly in Parkinson’s disease (Dehay et al., 2013), the toxic accumulation of misfolded proteins and subsequent neurodegeneration has been linked to lysosomal dysfunction (Dehay et al., 2010), (Lie and Nixon, 2019). Approaches to modulate lysosomal pH are scarce. Developing therapeutic nanosystems with acidic drug delivery to restore lysosomal acidification are of significant interest (Zeng et al., 2020). The main issue is to target neuronal lysosomes in neurodegenerative diseases to transport therapeutic agents to the central nervous system (CNS) and then pass through the Blood-Brain-Barrier (BBB). The size of nanoobjects and their lipophilic nature are crucial to cross this natural protective membrane (Zhang et al., 2009), (Rathore et al., 2019), (Edelmann and Maegawa, 2020). Polymeric or lipid nanoparticles (NPs) were developed to exhibit specific characteristics allowing the uptake into CNS. Mainly, PLGA polyester nanoparticles (diameter∼100 nm) are used to target CNS (Cunha et al., 2021). This pH and enzyme-sensitive polymer releases lactic and glycolic acid residues in a mild acid aqueous environment and has been reported to decrease lysosomal pH efficiently (Bourdenx et al., 2016), (Zeng et al., 2019). To achieve CNS targeting by systemic injection and overcome the low diffusion power of PLGA NPs in the brain, the development of oil-in-water (O/W) nanoemulsions (NEs)-based formulation was recently reported (Prévot et al., 2018). Potential of NEs for drug delivery to the brain was recently reviewed (Karami et al., 2019), highlighting the essential properties of NEs to this goal for hydrophobic therapeutics agents entrapped inside oil droplets, i.e., cellular transport either by paracellular or transcellular routes, the colloidal dispersion protecting them from both chemical and enzymatic degradations. Considering these properties and their biocompatibility based on excipients, which may be selected as Generally Recognized As Safe (GRAS), allowing their administration in humans, NE represents an excellent nanosystem for drug delivery and sustained drug release.

Nevertheless, the low solubility of PLGA polymer in oil and its limited loading rate into resulting NE led us to design and synthesize a small low molecular weight molecule able to be efficiently loaded into NE. Nucleolipids (NLs) are small hybrid bioinspired molecules composed of a lipid covalently linked to a nucleic acid derivative, nucleobase, nucleoside, nucleotide, or oligonucleotide with a certain structural similarity to cell membranes (Gissot et al., 2008). It is widely accepted that NLs are efficient molecular carriers for the delivery of therapeutic ingredients (Simeone et al., 2011), (Benizri et al., 2018), (Zhou et al., 2020). Their ability to cross the BBB was also recently highlighted (Swastika et al., 2019), making them good candidates for our goal. Based on this outcome, it was first demonstrated that a NL appropriately functionalized could be loaded in an O/W NE and subsequently efficiently internalized within human neuronal cells and co-localized with targeted lysosomes (Cunha et al., 2020).

**FIGURE 1:**
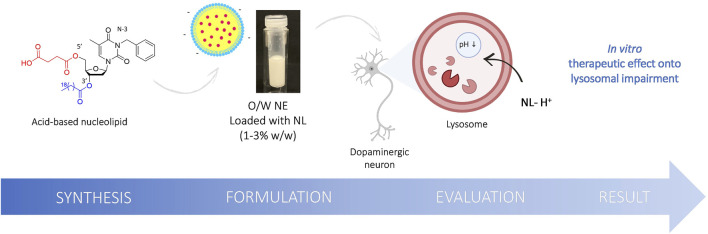
Schematic illustration of the structure of the original NL 8 bearing succinic acid moiety at the 5′ position, the formulation into O/W NE and the evaluation of the acidic NEs rescue lysosomal pH *in vitro*.

To go further, we report here the synthesis, the formulation, and the biological evaluation of original NLs designed as prodrugs and carrying an organic acid moiety as a potential lysosomal acidifying agent (Figure 1). Succinic acid was chosen on its excellent biocompatibility and its pKa range remarkably close to lactic and glycolic acids pKas. The free acid function was expected to provide rapid acidification. In contrast, the second acid function, protected as an ester at the 5′ position, will be released in a second time *via* enzymatic cleavage. Their formulation into O/W NEs was performed to allow the plasma membrane crossing. The nature of the fatty chain at the 3′ position was modulated to study the effect of the lipid chain length on cytotoxicity and acidifying capacity. The encouraging results reported herein suggest that O/W NEs loaded with acid NLs are efficient nanocarriers for drug delivery. Indeed, it has been shown that acidic NLs are easily transported to the lysosomes and rescue efficiently the impaired pH.

## Materials and Methods

### Synthesis and Characterization of Acidic Nucleolipids 7-8

#### General Methods

^1^H NMR and ^13^C NMR were recorded on a Bruker Avance 300 (^1^H: 300 MHz, ^13^C: 75.46 MHz) spectrometer using residual CHCl_3_ as an internal reference (7.26 ppm) and at 293 K unless otherwise indicated. The chemical shifts (δ) and coupling constants (*J*) are expressed in ppm and Hz. The following abbreviations were used to explain the multiplicities: s = singlet, d = doublet, t = triplet, q = quartet and m = multiplet. Fourier transform infrared (FT-IR) spectra were recorded on a PerkinElmer FT spectrometer Spectrum two (UATR two). For electrospray ionization (ESI) high-resolution mass spectrometry (HRMS) analyses, a Waters Micromass ZQ instrument equipped with an electrospray source was used in the positive and/or negative mode. Matrix-assisted laser desorption ionization time-of-flight (MALDI-TOF) mass spectrometric analyses were performed on a PerSeptive Biosystems Voyager-De Pro MALDI mass spectrometer in the linear mode using 3,4-dihydroxybenzoic acid as the matrix. Analytical thin-layer chromatography was performed using silica gel 60 F254 precoated plates (Merck) with visualization by ultraviolet light, potassium permanganate, or sulfuric acid. Flash chromatography was performed on a silica gel (0.043–0.063 mm).

#### Synthesis of Compound 1

In a vial, to a solution of thymidine (1 eq., 1 g, 4.13 mmol) in anhydrous DMF (10 ml) and under argon atmosphere, NaH (1 eq., 0.166 g) was added before a microwave activation (6 min, 40°C, 200 W). Then, benzyl bromide (1 eq., 490 μL) was added, and the reaction mixture was heated in a microwave (60 min, 40°C, 200 W). DMF was then evaporated with toluene without any further treatment. The reaction was repeated three times, the crudes were collected and purified by flash chromatography over silica gel (DCM/MeOH: 95/5) to afford the compound 1 as an expended solid (3.9 g, 95 %).

R_f_ = 0.4 (DCM/MeOH: 95/5). IR (ATR) νmax (cm^−1^) 3410, 2924, 2861, 1947, 1650, 1563, 1392, 1256, 1095, 655, 488. ^1^H-NMR (300 MHz, CD_3_OD) δ (ppm) 7.86 (d, *J* = 1.2 Hz, 1H), 7.38–7.17 (m, 5H), 6.31 (appearing t, *J* = 6.9, 13.5 Hz, 1H), 5.08 (brs, 2H), 4.43–4.35 (m, 1H), 3.94–3.87 (m, 1H), 3.76 (ddd, *J* = 2.7, 11.4, 23.1 Hz, 2H), 2.32–2.13 (m, 2H), 1.91 (s, 3H). ^13^C-NMR (75.5 MHz, CD_3_OD) δ (ppm) 165.3, 152.4, 138.3, 136.6, 129.4, 129.3, 128.4, 110.7, 88.9, 87.1, 72.1, 62.7, 45.3, 41.3, 13.2. HRMS (ESI): Calcd. for C_17_H_20_N_2_O_5_ [M + H]^+^ 333.13722, found 333.14470.

#### Synthesis of Compound 2

To a solution of compound 1 (1 eq., 3.18 g, 9.53 mmol) into dry pyridine (65 ml), were added under argon *t*-Butyldimethylsilyl chloride (1.2 eq., 1.73 g, 11.2 mmol) and the DMAP (0.1 eq., 116 mg, 0.953 mmol). The reaction mixture was stirred overnight at room temperature and then quenched with water (30 ml). The aqueous phase was extracted three times with DCM (3 × 30 ml), and the combined organic phases were dried over Na_2_SO_4_ before concentration under reduced pressure. The crude product was purified by flash chromatography over silica gel (Pentane/EtOAc: 70/30) to afford compound 2 as an expended solid (1.78 g, 89 %).

R_f_ = 0.3 (Pentane/EtOAc: 70/30). ^1^H-NMR (300 MHz, CDCl_3_) δ (ppm) 7.54–7.45 (m, 3H), 7.34–7.22 (m, 3H), 6.41 (dd, *J* = 5.7, 7.8 Hz, 1H), 5.17–5.10 (brs, 2H), 4.50–4.42 (m, 1H), 4.06–3.99 (m, 1H), 3.90 (dd, *J* = 2.7, 11.4 Hz, 1H), 3.82 (dd, *J* = 2.7, 11.4 Hz, 1H), 2.38 (ddd, *J* = 2.4, 5.7, 13.2 Hz, 1H), 2.16–2.04 (m, 1H), 1.95 (s, 3H), 0.93 (s, 9H), 0.13 (s, 3H), 0.12 (s, 3H). ^13^C-NMR (75.5 MHz, CDCl_3_) δ (ppm) 163.6, 151.1, 137.0, 133.7, 129.2, 128.5, 127.7, 110.3, 87.0, 85.7, 72.6, 63.6, 44.6, 41.4, 26.0, 18.5, 13.5, −5.3, −5.4. Analytical data are consistent with the literature (Cunha et al., 2020).

#### Synthesis of Compound 3

To a solution of compound 2 (1 eq., 0.718 g, 1.82 mmol) into dry DCM (10 ml), was added under argon the palmitic acid (1.2 eq., 0.560 g, 2.18 mmol), the EDC. HCl (1.2 eq., 0.419 g, 2.18 mmol) and the DMAP (0.5 eq., 0.111 g, 0.90 mmol). The reaction mixture was stirred at room temperature for 48 h and then quenched with water (10 ml). The aqueous phase was extracted three times with DCM (3 × 30 ml), and the combined organic phases were dried over Na_2_SO_4_ before concentration under reduced pressure. The crude product was purified by flash chromatography over silica gel (Pentane/EtOAc: 70/30) to provide compound 3 as a white foam (1.19 g, quantitative yield).

R_f_ = 0.9 (Pentane/EtOAc: 70/30) ^1^H-NMR (300 MHz, CDCl_3_) δ (ppm) 7.54 (d, *J* = 1.2 Hz, 1H), 7.52–7.47 (m, 2H), 7.35–7.22 (m, 3H), 6.42 (dd, *J* = 5.1, 9.3 Hz, 1H), 5.30–5.23 (m, 1H), 5.19–5.09 (m, 2H), 4.10–4.05 (m, 1H), 3.98–3.86 (m, 2H), 2.40 (dd, *J* = 5.4, 13.8 Hz, 1H), 2.33 (t, *J* = 7.5 Hz, 2H), 2.17–2.07 (m, 1H), 1.96 (d, *J* = 0.9 Hz, 3H), 1.70–1.57 (m, 2H), 1.38–1.21 (m, 26H), 0.94 (s, 9H), 0.98–0.85 (m, 3H), 0.14 (s, 3H), 0.14 (s, 3H). ^13^C-NMR (75.5 MHz, CDCl_3_) δ (ppm) 173.6, 163.4, 151.1, 137.0, 133.3, 129.3, 128.4, 127.6, 110.5, 85.5, 75.3, 63.7, 44.6, 38.1, 34.3, 32.0, 29.8, 29.7, 29.5, 29.4, 29.3, 29.2, 26.0, 24.9, 22.8, 18.4, 14.2, 13.3, −5.3, −5.4. Analytical Data Are Consistent With the Literature (Cunha et al., 2020).

#### Synthesis of Compound 5

To a solution of compound 3 (1 eq., 1.39 g, 2.03 mmol) into dry THF (20 ml) was added at 0°C, under argon the TBAF (1.2 eq., 0.590 ml, 2.03 mmol). The reaction mixture was stirred at 0°C for 3 h and then quenched with water (20 ml). The aqueous phase was extracted three times with DCM (3 × 30 ml) and the combined organic phases were dried over Na_2_SO_4_ before concentration under reduced pressure. The crude product was purified by flash chromatography over silica gel (Pentane/EtOAc: 70/30) to provide compound 5 as a white foam (0.821 g, 71%).

R_f_ = 0.3 (Pentane/EtOAc: 70/30). ^1^H-NMR (300 MHz, CDCl_3_) δ (ppm) 7.59 (d, *J* = 1.2 Hz, 1H), 7.50–7.44 (m, 2H), 7.33–7.21 (m, 3H), 6.31 (t, *J* = 7.2 Hz, 1H), 5.37–5.31 (m, 1H), 5.18–5.07 (m, 2H), 4.09–4.04 (m, 1H), 3.94–3.83 (m, 2H), 3.04 (brs, 1H), 2.41–2.28 (m, 4H), 1.92 (d, *J* = 0.6 Hz, 3H), 1.69–1.54 (m, 2H), 1.40–1.15 (m, 26H), 0.94–0.85 (m, 3H).

^13^C-NMR (75.5 MHz, CDCl_3_) δ (ppm) 173.7, 163.5, 151.1, 136.8, 134.5, 129.2, 128.5, 127.7, 110.7, 86.6, 85.2, 74.7, 62.7, 44.6, 37.4, 34.2, 32.1, 29.8, 29.7, 29.7, 29.5, 29.4, 29.3, 29.2, 24.9, 22.8, 14.2, 13.5. Analytical Data Are Consistent With the Literature (Cunha et al., 2020).

#### Synthesis of Compound 7

To a suspension of nucleolipid 5 (1eq., 500 mg, 0.88 mmol) into dry Toluene (10 ml), were added under argon the succinic anhydride (1eq., 88 mg, 0.88 mmol) and the DMAP (0.1 eq., 10 mg, 0.088 mmol). The reaction mixture was heated at 140°C and stirred for 2 h before concentration under reduced pressure. The crude product was purified by flash chromatography over silica gel (Toluene/EtOAc: 80/20) to provide compound 7 as a colorless gum (350 mg, 60%).

R_f_ = 0.1 (Toluene/Acetone: 80/20). IR (ATR) νmax (cm^−1^) 2924, 2857, 1835, 1717, 1649, 1563, 1455, 1357, 1254, 1159, 1100, 1004, 926, 828, 752, 706, 620, 534. ^1^H-NMR (300 MHz, CDCl_3_) δ (ppm) 7.54–7.41 (m, 2H), 7.36–7.19 (m, 4H), 6.33 (dd, *J* = 5.7, 8.7 Hz, 1H), 5.25–5.04 (m, 3H), 4.38 (ddd, *J* = 4.5, 12.3, 16.2 Hz, 1H), 4.27-4.15 (m, 1H), 2.77–2.58 (m, 4H), 2.44 (dd, *J* = 5.4, 14.1 Hz, 1H), 2.31 (appearing t, *J* = 7.2, 7.8 Hz, 2H), 2.21–2.05 (m, 1H), 1.93 (brs, 3H), 1.69–1.54 (m, 2H), 1.39–1.18 (m, 24H), 0.95–0.81 (m, 3H). ^13^C-NMR (75.5 MHz, CDCl_3_) δ (ppm) 177.1, 173.5, 171.8, 163.4, 151.0, 136.8, 132.9, 129.3, 128.5, 127.7, 110.9, 85.6, 82.2, 74.0, 64.2, 44.7, 37.6, 34.2, 32.0, 29.8, 29.7, 29.5, 28.8, 28.7, 24.9, 22.8, 14.2, 13.5. HRMS (ESI): Calcd. for C_37_H_54_N_2_O_9_ [M + H]^+^ 671.38293, found 671.39051.

#### Synthesis of Compound 4

To a solution of compound 2 (1 eq., 2.1 g, 5.3 mmol) into dry DCM (30 ml), was added under argon the arachidic acid (1.5 eq., 2.5, 8.0 mmol) the EDC. HCl (1.5 eq., 1.53 g, 8.0 mmol) and the DMAP (0.7 eq., 0.46 g, 3.7 mmol). The reaction mixture was stirred at room temperature for 48 h and then quenched with water (30 ml). The aqueous phase was extracted three times with DCM (3 × 30 ml), and the combined organic phases were dried over Na_2_SO_4_ before concentration under reduced pressure. The crude product was purified by flash chromatography over silica gel (Pentane/EtOAc: 70/30) to provide 4 as a white foam (3.64 g, quantitative yield).

R_f_ = 0.9 (Pentane/EtOAc: 70/30). IR (ATR) νmax (cm^−1^) 2926, 2855, 2254, 1736, 1703, 1668, 1645, 1496, 1464, 1404, 1352, 1321, 1294, 1259, 1166, 1126, 1079, 1004, 903, 833, 780, 701, 648, 616, 536, 495, 465. ^1^H-NMR (300 MHz, CDCl_3_) δ (ppm) 7.55 (d, *J* = 0.9 Hz, 1H), 7.59–7.47 (m, 2H), 7.36–7.22 (m, 3H), 6.42 (dd, *J* = 5.1, 9.3 Hz, 1H), 5.29–5.23 (m, 1H), 5.21–5.08 (m, 2H), 4.11–4.06 (m, 1H), 3.98–3.86 (m, 2H), 2.41 (dd, *J* = 5.4, 13.8 Hz, 1H), 2.33 (t, *J* = 7.5 Hz, 2H), 2.18–2.03 (m, 1H), 1.96 (d, *J* = 0.9 Hz, 3H), 1. 71–1.57 (m, 2H), 1.39–1.22 (m, 32H), 0.94 (s, 9H), 0.97–0.85 (m, 3H), 0.14 (s, 3H), 0.14 (s, 3H). ^13^C-NMR (75.5 MHz, CDCl_3_) δ (ppm) 178.9, 173.6, 163.5, 151.1, 137.0, 133.3, 129.3, 128.5, 127.7, 110.6, 85.5, 75.3, 63.7, 44.6, 38.1, 34.3, 32.0, 29.8, 29.8, 29.7, 29.5, 29.5, 29.3, 29.2, 26.0, 24.9, 22.8, 18.4, 14.2, 13.4, - 5.3, −5.4. HRMS (ESI): Calcd. for C_43_H_72_N_2_O_6_Si [M + H]^+^ 741.51596, found 741.52431.

#### Synthesis of Compound 6

To a solution of compound 4 (1 eq., 1.28 g, 1.73 mmol) into dry THF (19 ml) was added at 0°C, under argon the TBAF (1.2 eq., 0.50 ml, 1.90 mmol). The reaction mixture was stirred at 0°C for 3 h and then quenched with water (20 ml). The aqueous phase was extracted three times with DCM (3 × 30 ml), and the combined organic phases were dried over Na_2_SO_4_ before concentration under reduced pressure. The crude product was purified by flash chromatography over silica gel (Pentane/EtOAc: 70/30) to provide the compound 6 as a white foam (720 mg, 70%).

R_f_ = 0.3 (Pentane/EtOAc: 7/3). IR (ATR) νmax (cm^−1^) 3403, 2918, 2852, 2057, 1987, 1938, 1832, 1786, 1711, 1635, 1461, 1358, 1235, 1170, 1096, 992, 928, 718, 615, 536, 448, 432, 407. ^1^H-NMR (300 MHz, CDCl_3_) δ (ppm) 7.67–7.62 (m, 1H), 7.49–7.41 (m, 2H), 7.33–7.17 (m, 3H), 6.32 (appearing t, *J* = 7.5, 6.6 Hz, 1H), 5.37–5.30 (m, 1H), 5.11 (s, 2H), 4.08–4.02 (m, 1H), 3.89–3.83 (m, 2H), 3.54 (brs, 1H), 2.38–2.26 (m, 2H), 1.89 (brs, 3H), 1.68–1.56 (m, 2H), 1.35–1.22 (m, 32H), 0.92–0.83 (m, 3H). ^13^C-NMR (75.5 MHz, CDCl_3_) δ (ppm) 173.6, 163.5, 151.0, 136.7, 134.6, 129.0, 128.3, 127.6, 110.3, 86.3, 85.2, 74.8, 62.4, 44.5, 31.9, 29.7, 29.6, 29.6, 29.4, 29.3, 29.2, 29.1, 24.8, 22.7, 14.1, 13.3. HRMS (ESI): Calcd. for C_37_H_58_N_2_O_6_ [M + H]^+^ 627.42949, found 627.43718.

#### Synthesis of Compound 8

To a suspension of nucleolipid 6 (1 eq., 100 mg, 0.17 mmol) into dry toluene (10 ml), were added under argon the succinic anhydride (1 eq, 17 mg, 0.17 mmol) and the DMAP (0.1 eq., 2 mg, 0.017 mmol). The reaction was heated at 140°C and stirred for 4 h before concentration under reduced pressure. The crude product was purified by flash chromatography over silica gel (Toluene/EtOAc: 80/20) to provide 8 as a colorless gum (75 mg, 66%).

R_f_ = 0.1 (Toluene/EtOAc: 8/2). IR (ATR) νmax (cm^−1^) 2923, 2853, 1738, 1705, 1668, 1645, 1496, 1454, 1352, 1317, 1272, 1247, 1157, 1102, 1078, 1002, 909, 826, 766, 732, 700, 618, 538, 495, 466, 421. ^1^H-NMR (300 MHz, CDCl_3_) δ (ppm) 7.54–7.43 (m, 2H), 7.38–7.22 (m, 4H), 6.33 (dd, *J* = 5.7, 8.7 Hz, 1H), 5.26–5.06 (m, 3H), 4.37 (ddd, *J* = 4.5, 12.3, 30.9 Hz, 2H), 4.24-4.16 (m, 1H), 2.77–2.59 (m, 4H), 2.43 (dd, *J* = 5.4, 14.1 Hz, 1H), 2.31 (t, *J* = 7.5 Hz, 2H), 2.21–2.06 (m, 1H), 1.94 (brs, 3H), 1.71–1.53 (m, 2H), 1.41–1.18 (m, 34H), 0.93–0.81 (m, 3H); ^13^C-NMR (75.5 MHz, CDCl_3_) δ (ppm) 177.1, 173.5, 171.8, 163.4, 151.0, 136.9, 132.9, 129.3, 128.5, 127.8, 111.0, 85.6, 82.3, 74.0, 64.2, 44.7, 37.6, 34.2, 32.1, 29.8, 29.8, 29.7, 29.6, 29.5, 29.4, 29.2, 28.8, 28.6, 24.9, 22.8, 14.3, 13.5. HRMS (ESI): Calcd. for C_41_H_62_N_2_O_9_ [M + H] + 727.44553, found 727.45319.

### Nanoemulsion Preparation and Characterization

#### NEs Formulation

For all experiments, O/W NE were used as freshly prepared, formulated with medium chain triglyceride oil (Miglyol® 812N) and two surfactants. To obtain the oily phase, 12 mg egg lecithin (Lipoid® E80) were dispersed in 200 mg 70°C heated oil phase Miglyol® 812N. The aqueous phase was composed of a dispersion of 25 mg Polysorbate 80 (Tween® 80) in the 70°C heated 800 mg Milli-Q water. NE-5 to NE-8 were loaded respectively with compounds 5 to 8, solubilized at various final concentrations in NEs at 1.8 × 10^4^ μM for NL 5; 1.6 × 10^4^ μM for NL 6; 1.5, 4.5 and 10.5 × 10^4^ μM for NL 7; 1.4 and 4.2 × 10^4^ μM for NL 8. To adjust osmolality for *in vitro* experiments, Glycerol at 2.25% was added to the aqueous phase. Phase inversion, with previously prepared and heated phases and homogenization by sonication (Sonic Vibra Cell-VC 250), afforded the emulsion with submicron size range oil droplets. NEs typical composition in % w/w is given in Table 1.

**TABLE 1:** Composition of NEs loaded with acid-based NLs.

	NE-NL
NL content (%) w/w^a^	1–7%
Lipoïd^®^ E80	1.2%
Miglyol^®^ 812N	20%
Tween^®^ 80	2.5%
Glycerol	2.25%
H_2_O *qs*	100%

a NLs final concentrations were set at 1% and, depending on their solubility in the oil phase, were set additionally at 3 and 7% for NL 7 and 3% for NL 8.

#### NEs Characterization

Physical characteristics of all NEs were assessed by dynamic light scattering (DLS) using Malvern Instruments (Zetasizer Nano ZS). NEs were diluted at 1/2500 (v/v), and the average size and the polydispersity index were determined by three independent measurements performed at 25°C. To analyze the ζ-potential, NEs were diluted at 1/1500 (v/v), and measurements were performed using Zetasizer Nano ZS coupled with a folded capillary cell (DTS1060) from Malvern Instruments. Colloidal stability of loaded NEs was demonstrated for at least 3 months. The droplet’s mean diameter, index of polydispersity, and ζ-potential remained stable during the study. The macroscopic aspect showed no phase separation nor creaming during all study time.

### Cell Culture and Cell Viability Assay of Nucleolipid-Loaded Nanoemulsions

Human neuroblastoma cell lines BE(2)-M17 were obtained from ATCC (CRL-2267) (Filograna et al., 2015) and grown in OPTIMEM (Life Technologies, 31985–047) plus 10% fetal bovine serum (Sigma-Aldrich). ATP13A2 stable knockdown BE-(2)-M17 (shATP13A2(403–1)) human dopaminergic neuroblastoma cells were maintained at 37°C in 5% CO_2_ in OPTIMEM supplemented with 10% fetal calf serum, 1% penicillin/streptomycin, and 2 mg/ml puromycin (Sigma-Aldrich). For NE treatments, cells were grown at 70–80% confluence and treated for 24 h with 1 μL of NE diluted to 1/1000. Each experiment was reproduced at least in three independent series. Cell viability was estimated by MTT assay (ATCC/LGC Promochem) following manufacturer instructions.

### Lysosomal pH Measurement

Quantification of lysosomal pH was determined using dextran conjugates LysoSensor Yellow/Blue DND-160 (Life Technologies) and was performed as previously described (Bourdenx et al., 2016; Dehay et al., 2012). Briefly, control and mutant ATP13A2 cells were grown in their respective media. Cells were then trypsinized, harvested (1 × 10^6^ cells/mL), and loaded with 1 mg/ml of LysoSensor-dextran for 1 h at 37°C with 5% CO_2_. The cells were then washed 3 × in HBSS (Gibco, 14060) and aliquoted at 100 ml into a black 96-well microplate. pH calibration was performed as previously described. Wild-type and mutant cells were treated with 10 mM monensin (Sigma-Aldrich) and 10 mM nigericin (Sigma-Aldrich) in MES buffer (5 mM NaCl, 115 mM KCl, 1.3 mM MgSO_4_, 25 mM MES), with the pH adjusted to a range from 3.5 to 7.0. The samples were read in a FLUOstar Optima fluorimeter (BMG Labtech, Champigny sur Marne, France) with excitation at 355 nm. The ratio of emission 440/535 nm was then calculated for each sample. The pH values were determined from the standard linear curve generated via the pH calibration samples.

## Results and Discussion

### Design and Synthesis of the Nucleolipid-Based Platform

**SCHEME 1:**
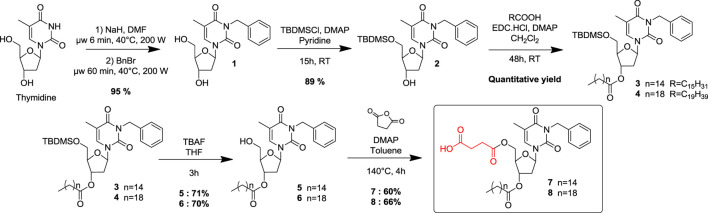
Synthetic pathway to generate NL 7-8.

Two original NLs (compounds 7 and 8) bearing a succinic acid moiety at the 5’ position and respectively palmitate and arachidate esters at the 3′ position, were designed, synthesized, and biologically evaluated as lysosomal pH modulators. Commercially available thymidine was used as a synthetic platform, and key positions were conveniently functionalized step-by-step (Scheme 1). Previous work has shown that a benzyl group, conveniently inserted at the N-3 position, increased sufficiently the lipophilicity to allow the compound to be solubilized in oil and then provide its subsequent incorporation into an O/W NE (Cunha et al., 2020). A benzyl group was then selectively introduced at the N-3 position of thymidine, using a microwave-assisted activation step as described in the literature (Hamoud et al., 2018). Then the hydroxyl at 5′ position was protected as a silylated ether. A classical esterification step with two different monocatenar fatty chains (palmitic, arachidic acids) at 3′ position provided compounds 3 and 4 in quantitative yields. The choice of the optimal length of the fatty chains was made in agreement with the previous results (Cunha et al., 2020). The silylated ether was then cleaved under classical conditions before the last step to functionalize the hydroxyl at 5′ as succinate. Various attempts to provide selective esterification with succinic acid became unsuccessful; succinate was then introduced by opening succinic anhydride under robust desymmetrization conditions (Catry and Madder, 2007). This straightforward five-step sequence provided efficiently acidic NLs 7-8 with fatty chains of 16 and 20 carbons, respectively. Under physiological conditions, compounds 7-8 should be cleaved to release the active succinic acid and regenerate the corresponding precursors 5 and 6, respectively. 5 and 6 are themselves also degradable, releasing palmitic and arachidic acid.

**SCHEME 2:**
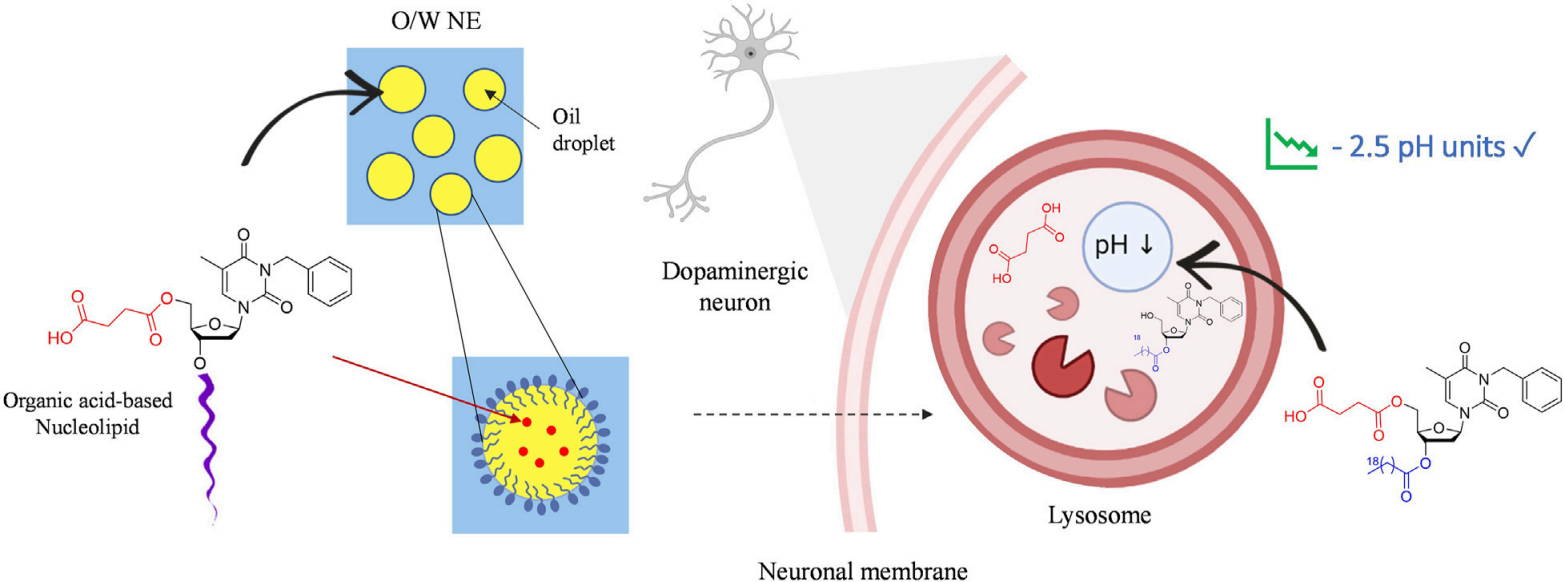
Graphical illustration of neuronal lysosomal acidification by nucleolipidic nanoemulsions.

O/W NEs have an excellent loading capacity for hydrophobic drugs while improving their stability compared to other lipidic formulations, i.e., liposomes used in clinics (Sainsbury et al., 2014). Indeed, the very small droplet size in the nanometric scale NEs makes them suitable for intravenous drug delivery. All NEs were formulated with 20% medium-chain triglyceride oil (Miglyol® 812N), 1.2% lecithin 80, 2.5% polysorbate 80 surfactant. An unloaded NE, NE-0 was used as a control. NLs acids 7-8 and intermediates 5-6 were respectively loaded in NEs NE-5, NE-6, NE-7, NE-8 with final concentrations from 1 to 7% (w/w), depending on their solubility in oil. The loading was successfully performed without altering the physical parameters, i.e., mean droplets size, granulometric profiles, and zeta potential, compared to the unloaded NE. However, a slight increase of the NE’s hydrodynamic mean diameter was observed for the highest concentrations of the loaded NLs for both NE-8 and NE-7 (data not shown). This modification did not alter the granulometric profile that stayed narrow with PDI below 0.2 or the system’s stability. Finally, compared to commonly commercialized intravenous drug-loaded NEs such as Propofol, exhibiting mean diameters between 250 and 280 nm, the slight increase of the hydrodynamic diameter did not alter the capacity of the system to be intravenously administered (Patil et al., 2019).

Analysis by DLS displayed an average diameter lower than 200 nm. This size is consistent for nanoobjects to be easily uptaken into cells and to colocalize subcellular organelles. A monodisperse distribution with a submicronic size range and ζ-potential values around −35 mV were also measured. Negative zeta potential is compatible with brain targeting and is recognized as non-toxic in comparison to cationic NEs (Lockman et al., 2004). Thus, negatively charged nanocarriers are privileged. All our formulations exhibited stable parameters for at least 3 months, as shown by compound 8 being the most effective (NE-8-1% was loaded with 1% w/w of NL 8, NE-8-3% was loaded with 3% w/w of NL 8). (Table 2).

**TABLE 2:** Physico-chemical characteristics of selected NEs: unloaded one, and those loaded with the effective NL.

	NE-0		NE-8-1%		NE-8-3%	
	T0	3 months	T0	3 months	T0	3 months
Droplets mean diameter (nm)	175.2 ± 1.36	175.9 ± 2.48	156.5 ± 2.71	155.6 ± 1.56	198.8 ± 2.65	193.6 ± 3.17
Polydispersity index	0.125	0.111	0.099	0.087	0.135	0.124
Zeta potential (mv)	−31.2 ± 1.8	−22.9 ± 0.1	−34.4 ± 1.0	−28.2 ± 2.3	−33.8 ± 0.8	−29.3 ± 1.2

### Acidic Nanoemulsions Rescue Lysosomal pH *in Vitro*

**FIGURE 2:**
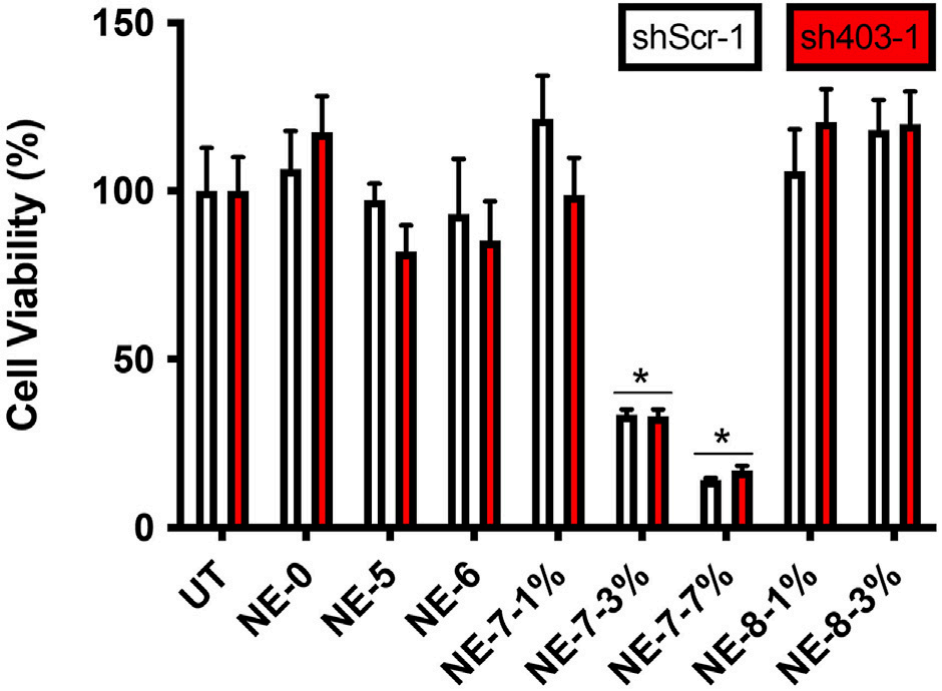
Cell viability in control (shScr-1) and ATP13A2-depleted (sh403-1) M17 cells, in the absence (UT) or presence of NE-0, NE-5, NE-6, NE-7 and NE-8 after 24 h *p < 0.05 compared with untreated cells.

Succinic acid is a biocompatible dicarboxylic acid present in all living organisms. This molecule is involved in cellular metabolism, particularly in the lipid metabolism. To investigate whether our NEs, loaded with our succinic-acid-based prodrugs at various concentrations, exhibit a cellular cytotoxicity, human neuroblastoma cells were exposed to them for 24 h. First, *in vitro* viability assays, with NEs diluted to 1/1000, confirmed that acidic NE-8 loaded with the arachidic derivative NL 8 (1 and 3% w/w concentrations) did not induce cell death for the loading rates tested (Figure 2). Moreover, NE-5 and NE-6, loaded respectively with 5 and 6, intermediates resulting from the partial degradation of the final prodrugs, did not induce any cytotoxicity either. However, the biological evaluation showed cytotoxicity for NE-7, bearing a palmitic acid chain at 3′ position, for the nanoemulsions loaded with 3 and 7% w/w of NL 7. It was not possible to perform viability assays with NE-5 and NE-6 at the same 3% w/w concentration as NE-7-3%, making it challenging to explain the cytotoxicity observed for NE-7. Further biological work will be necessary to raise this point. Thus, the results highlight the biocompatibility of our NEs, paving the way to test their effect on the lysosomal pH assessment.

**FIGURE 3:**
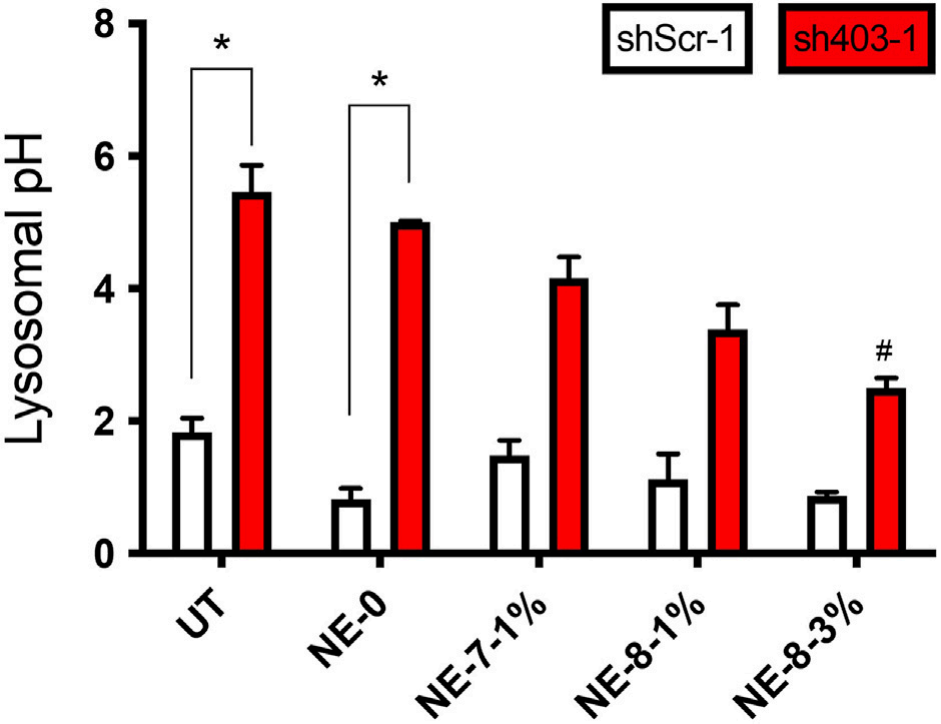
Acidic NEs rescue lysosomal pH *in vitro*. Lysosomal pH values in control (shScr-1) and ATP13A2-depleted (sh403-1) M17 cells, in the absence or presence of different NEs. *, *p* < 0.05 compared with shScr-1 untreated cells; #, *p* < 0.05 compared with sh403-1 untreated cells.

Lysosomal pH was assessed on M17 neuronal cell line stably depleted of ATP13A2, a lysosomal type 5 P-type ATPase. This cellular model displays abnormally high pH in their lysosomes since an impairment of ATP13A2 function increases lysosomal pH (Usenovic and Krainc, 2012; Dehay et al., 2013; Bento et al., 2016; Bourdenx et al., 2016). *In vitro* investigations on the effect of NL-NE on lysosomal pH highlighted that NE loaded with the longest fatty chains-based NLs turned out to be adequate to recover the acidic medium to normal levels (pH = 3), without lowering the lysosomal pH of control cells. The NE loaded incubated for 24 h, allowed a significant restoration of the lysosomal pH (Figure 3). It is assumed that the final acidification resulting from the *in vitro* degradation of the NL 7-8, is potentially due to the release of a mixture of the constitutive acids, including succinic acid. It looks like an active substance concentration of 7 µM starts to acidify the medium, while a concentration of 40 µM allows a total recovery of the lysosomal acidification defects. These results are promising for using such nanocarriers as tools to rescue lysosomal function *in vitro* in ATP13A2-knockdown cells with acidic NL-based NEs.

## Conclusion

A functional lysosome is crucial for maintaining proper cellular and neuronal function. Hence, targeting lysosomal impairment is becoming an increasingly important goal in developing treatments for many diseases including neurodegenerative diseases. In this context, restoring its dysfunctional acidic medium could provide a therapeutic option. To overcome the shortcomings of the current chemical arsenal mainly based on PLGA polymeric NPs, the first low molecular weight acid-based NL nanocarriers were developed to formulate and carry the biocompatible organic succinic diacid to the lysosome. A straightforward five-step sequence, starting from thymidine, afforded acidic NLs 7-8, bearing 16 and 20 carbons saturated fatty chains respectively in good overall yields. O/W NEs were loaded with NLs acids 7-8 and their precursors 5-6 to provide stable nanosystems: NE-5, NE-6, NE-7, NE-8. NEs’ formulation was designed with commonly used intravenous excipients to help the further transfer from laboratory bench side to patient bedside. *In vitro*, NE-8-3% (*c* = 40 µM of active substance), was shown not only to cross the membrane without any cytotoxicity but also to completely restore the acidic pH of the lysosome in a genetic cell model of Parkinson’s disease.

This very positive result, equivalent to that obtained with PLGA, shows that it is possible to overcome the limits of the use of this polymer. Varying the nature of the organic acid could provide a released acidification modulation. Moreover, the NL was developed as a synthetic platform that can be tuned to carry other active ingredients. Further *in vivo* studies will focus on the transposability of this innovative therapeutic approach and evaluate the ability of the DNA-based nanocarrier to cross the BBB. A positive outcome would allow us to consider NL functionalization with suitable active substances for other neurodegenerative diseases.

The authors declare that the research was conducted in the absence of any commercial or financial relationships that could be construed as a potential conflict of interest.

## Data Availability

The original contributions presented in the study are included in the article/Supplementary Material, further inquiries can be directed to the corresponding authors.
