# Supplementary material for: Nucleolipid Acid-Based Nanocarriers Restore Neuronal Lysosomal Acidification Defects
Source: Front Chem. 2021 Aug 20;9:736554. doi: 10.3389/fchem.2021.736554 (PMC8417785; doi:10.3389/fchem.2021.736554)
Supplement: Supplementary file 1 [file DataSheet2.pdf]

Nucleolipid acid-based nanocarriers restore neuronal lysosomal acidification defects

Mathias Brouillard[1], Philippe Barthélémy[1], Benjamin Dehay[2]*, Sylvie Crauste-Manciet[1,3], Valérie Desvergnes[1]*.

[1]University of Bordeaux, INSERM U1212, UMR CNRS 5320, F-33076 Bordeaux, France
[2] Univ. Bordeaux, CNRS, IMN, UMR 5293, F-33000 Bordeaux, France
[3]University Hospital, CHU Bordeaux, 33000 Bordeaux, France.

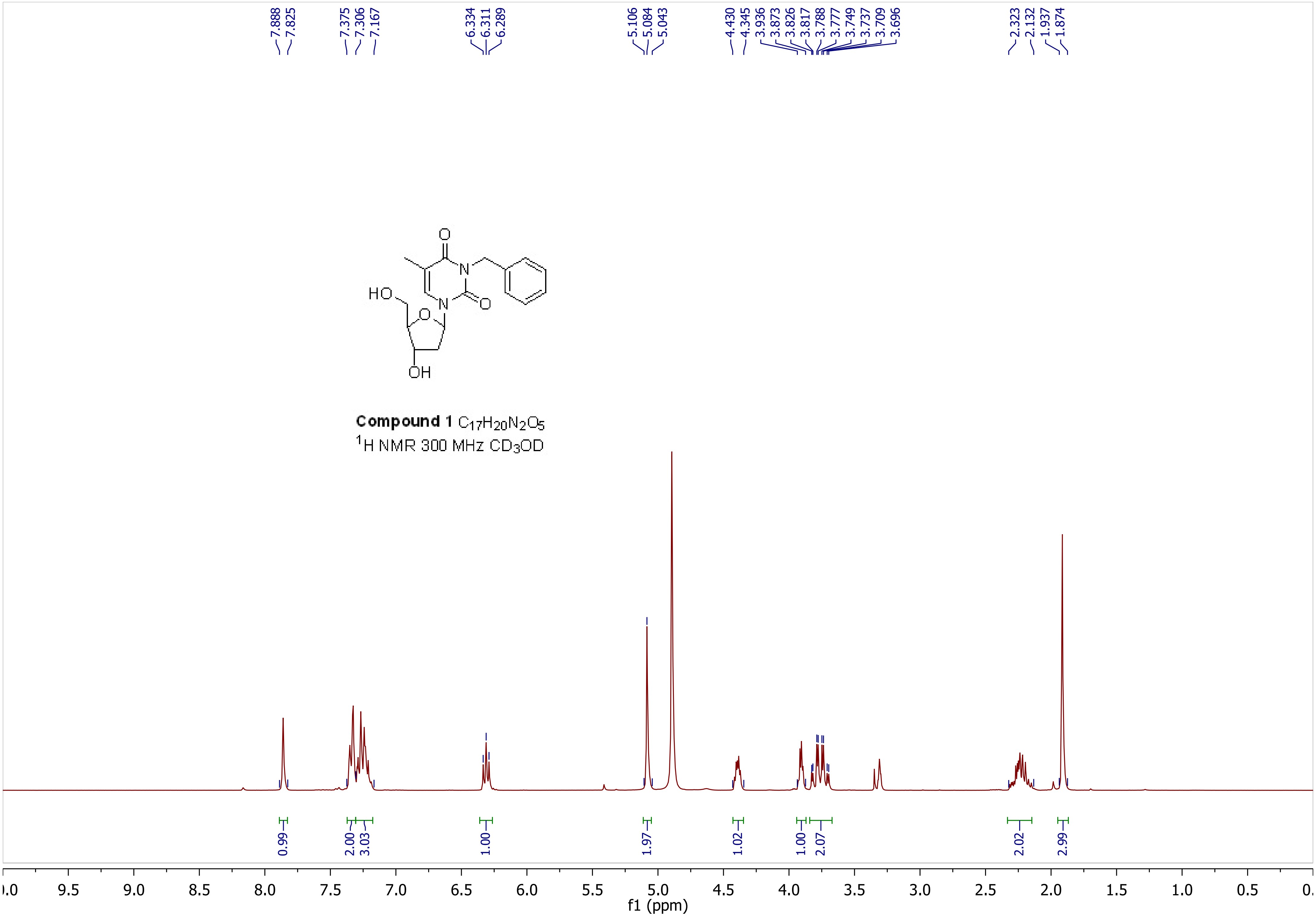
7.888
7.825
7.375
7.306
7.167
6.334
6.311
6.289
5.106
5.084
5.043
4.430
4.345
3.936
3.873
3.826
3.817
3.788
3.777
3.749
3.737
3.709
3.696
2.323
2.132
1.937
1.874
HO
O
N
N
O
O
OH
Compound 1 $C_{17}H_{20}N_2O_5$
^{1}H NMR 300 MHz CD_3OD
0.99
2.00
3.03
1.00
1.97
1.02
1.00
2.07
2.02
2.99
9.5
9.0
8.5
8.0
7.5
7.0
6.5
6.0
5.5
5.0
4.5
4.0
3.5
3.0
2.5
2.0
1.5
1.0
0.5
f1 (ppm)

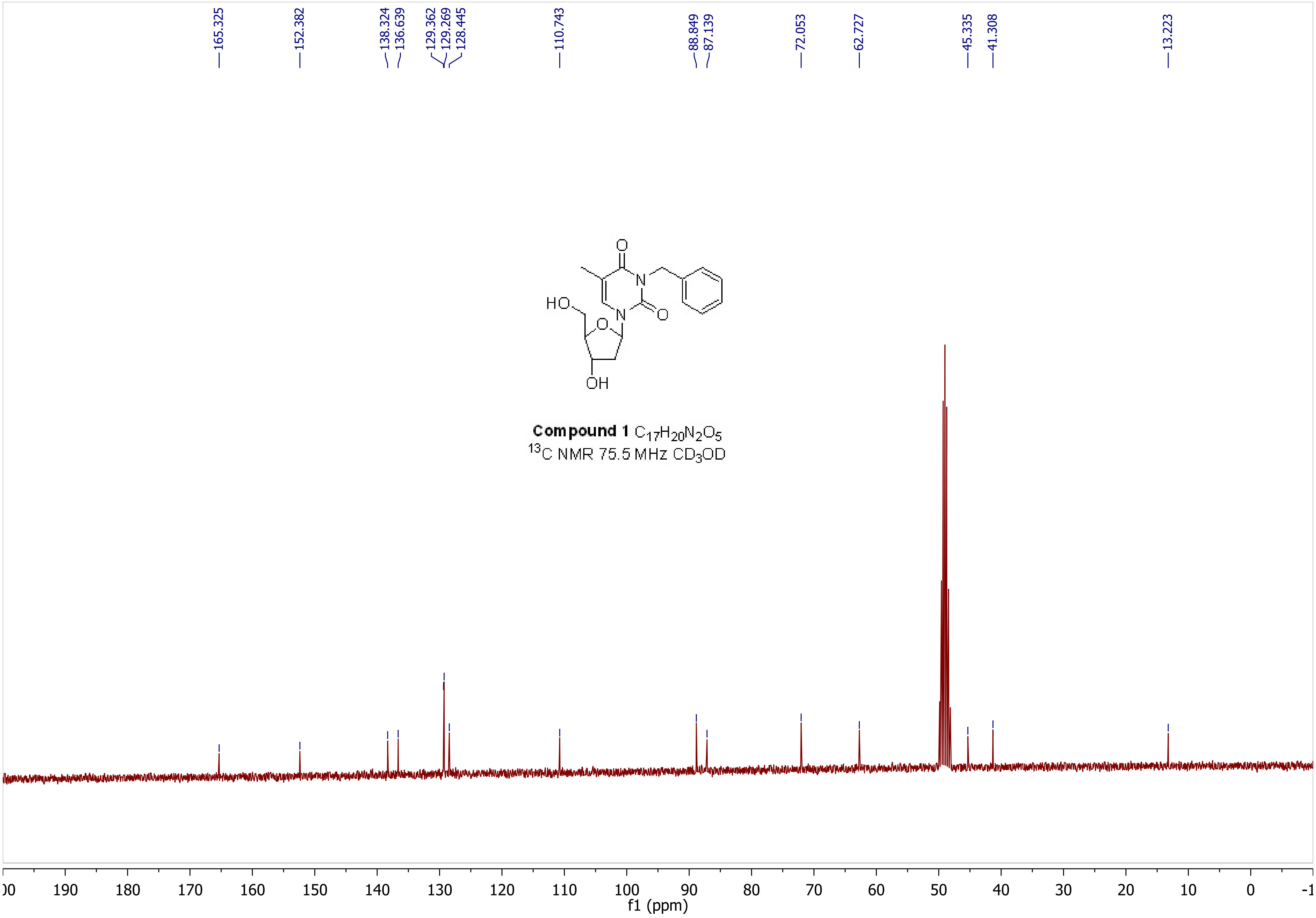
165.325
152.382
138.324
136.639
129.362
129.269
128.445
110.743
88.849
87.139
72.053
62.727
45.335
41.308
13.223
HO
O
N
OH
Compound 1 $C_{17}H_{20}N_2O_5$
^{13}C NMR 75.5 MHz CD_3OD
f1 (ppm)

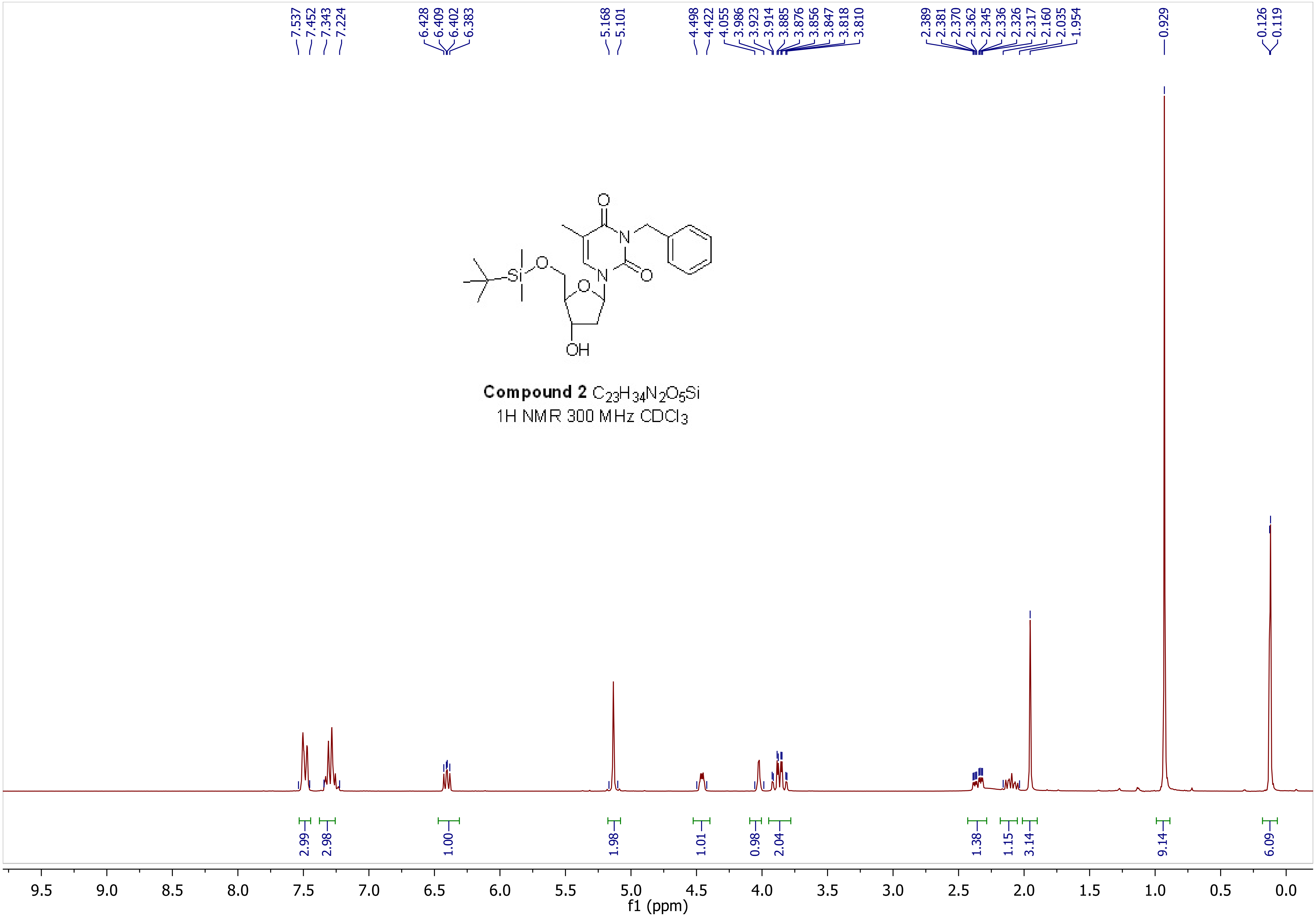
7.537
7.452
7.343
7.224
6.428
6.409
6.402
6.383
5.168
5.101
4.498
4.422
4.055
3.986
3.923
3.914
3.885
3.876
3.856
3.847
3.818
3.810
2.389
2.381
2.370
2.362
2.345
2.336
2.326
2.317
2.160
2.035
1.954
0.929
0.126
0.119
O
N
N
O
Si
O
O
OH
Compound 2 $C_{23}H_{34}N_2O_5Si$
1H NMR 300 MHz $CDCl_3$
2.99
2.98
1.00
1.98
1.01
0.98
2.04
1.38
1.15
3.14
9.14
6.09
9.5
9.0
8.5
8.0
7.5
7.0
6.5
6.0
5.5
5.0
4.5
4.0
3.5
3.0
2.5
2.0
1.5
1.0
0.5
0.0
f1 (ppm)

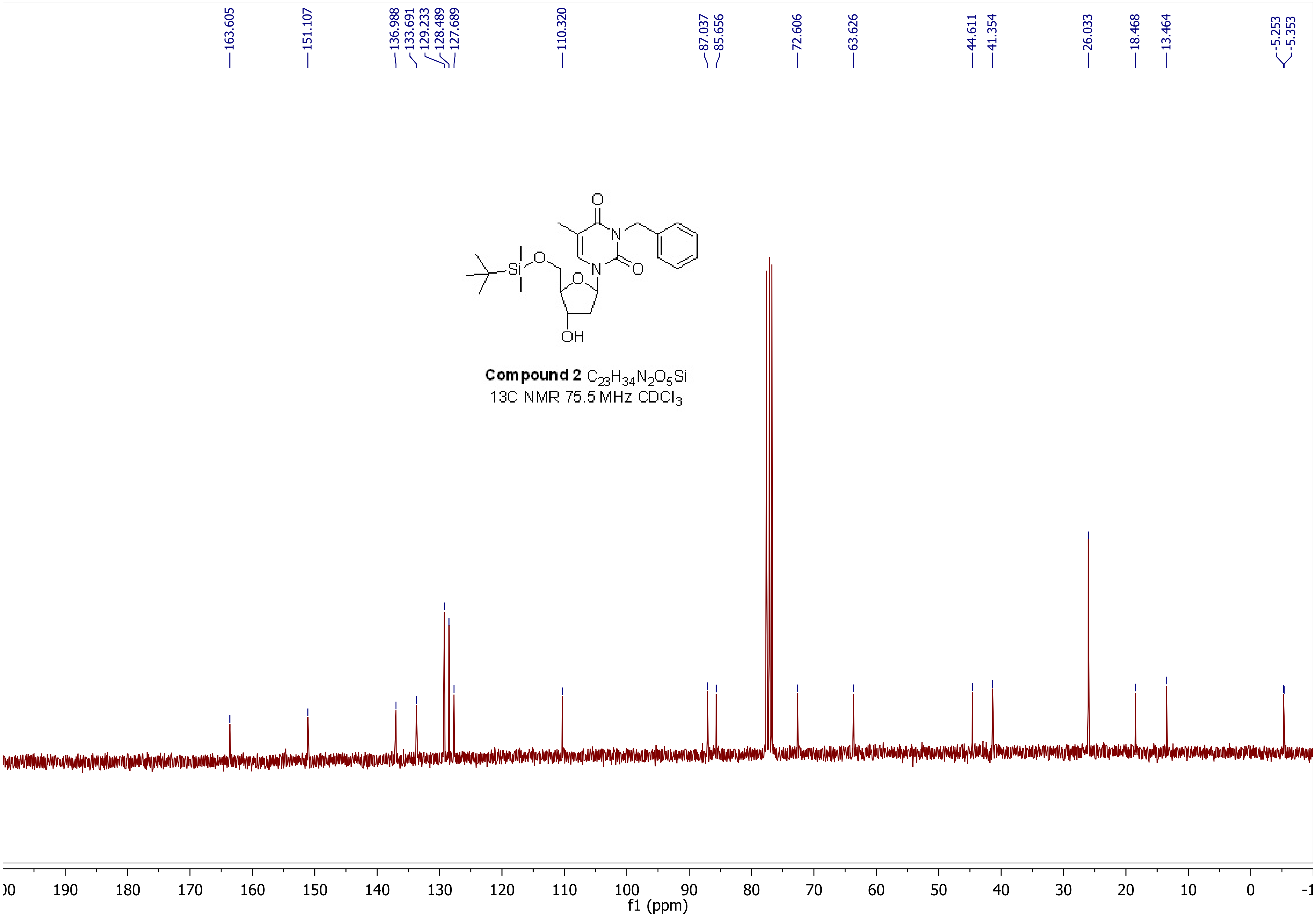
163.605
151.107
136.988
133.691
129.233
128.489
127.689
110.320
87.037
85.656
72.606
63.626
44.611
41.354
26.033
18.468
13.464
-5.253
-5.353
O
N
N
O
Si
O
O
OH
Compound 2 $C_{23}H_{34}N_2O_5Si$
13C NMR 75.5 MHz $CDCl_3$
00 190 180 170 160 150 140 130 120 110 100 90 80 70 60 50 40 30 20 10 0 -1
f1 (ppm)

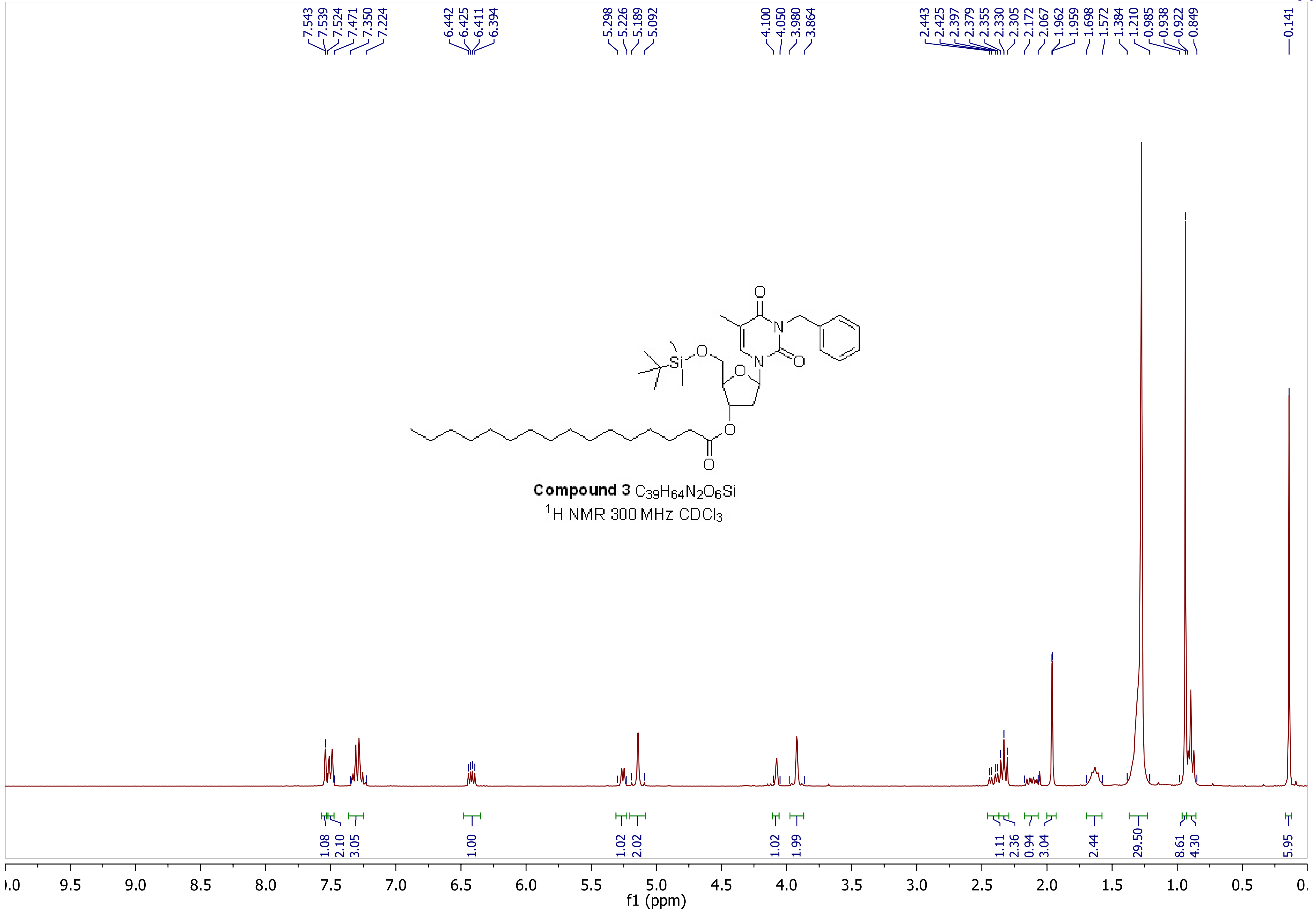
7.543 7.539 7.524 7.471 7.350 7.224
6.442 6.425 6.411 6.394
5.298 5.226 5.189 5.092
4.100 4.050 3.980 3.864
2.443 2.425 2.397 2.379 2.355 2.330 2.305 2.172 2.067 1.962 1.959 1.698 1.572 1.384 1.210 0.985 0.938 0.922 0.849
0.141
Compound 3 C39H64N2O6Si
1H NMR 300 MHz CDCl3
1.08 2.10 3.05 1.00 1.02 2.02 1.02 1.99 1.11 2.36 0.94 3.04 2.44 29.50 8.61 4.30 5.95
9.5 9.0 8.5 8.0 7.5 7.0 6.5 6.0 5.5 5.0 4.5 4.0 3.5 3.0 2.5 2.0 1.5 1.0 0.5
f1 (ppm)

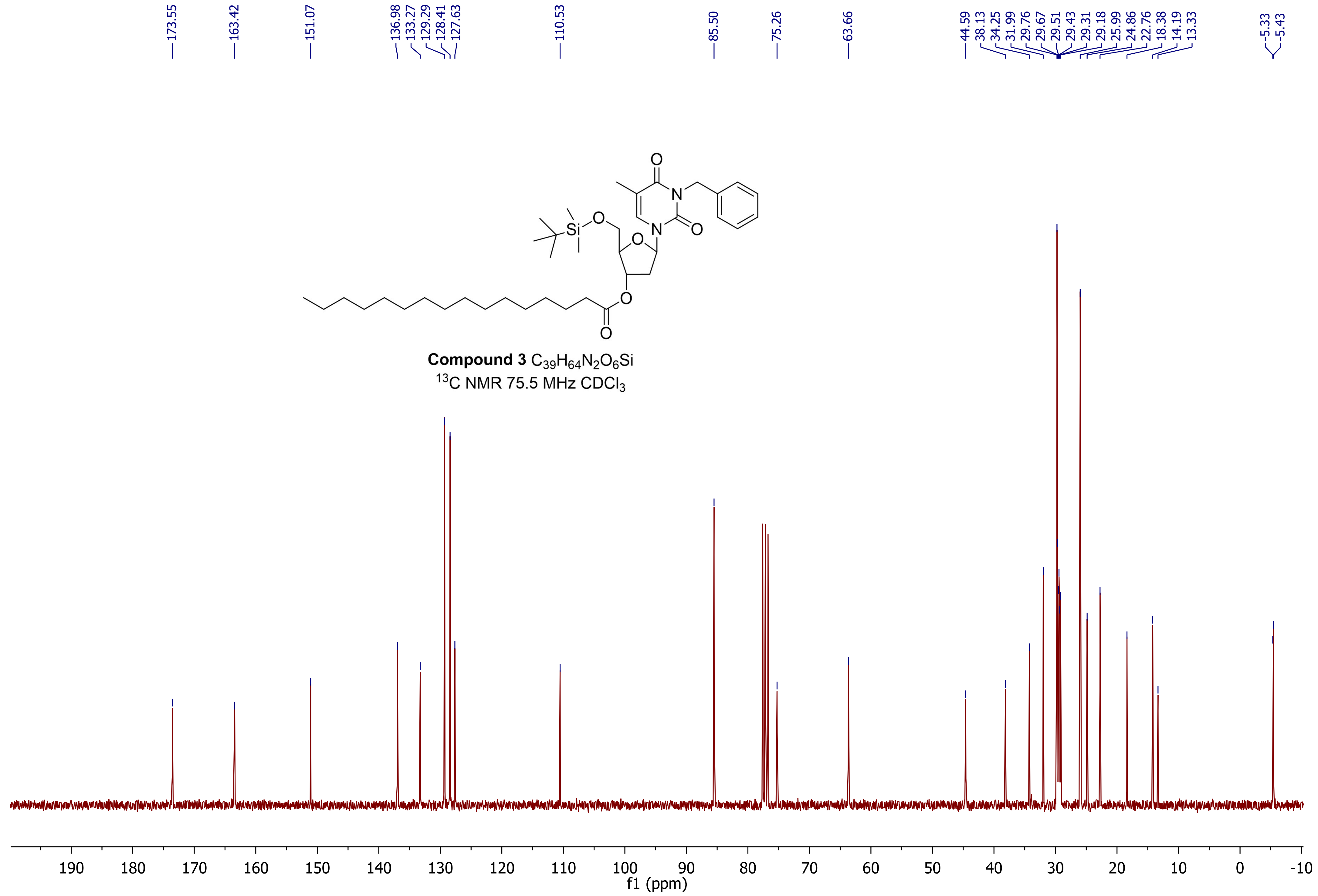
173.55
163.42
151.07
136.98
133.27
129.29
128.41
127.63
110.53
85.50
75.26
63.66
44.59
38.13
34.25
31.99
29.76
29.67
29.51
29.43
29.31
29.18
25.99
24.86
22.76
18.38
14.19
13.33
-5.33
-5.43
Compound 3 $C_{39}H_{64}N_2O_6Si$
^{13}C NMR 75.5 MHz $CDCl_3$
f1 (ppm)

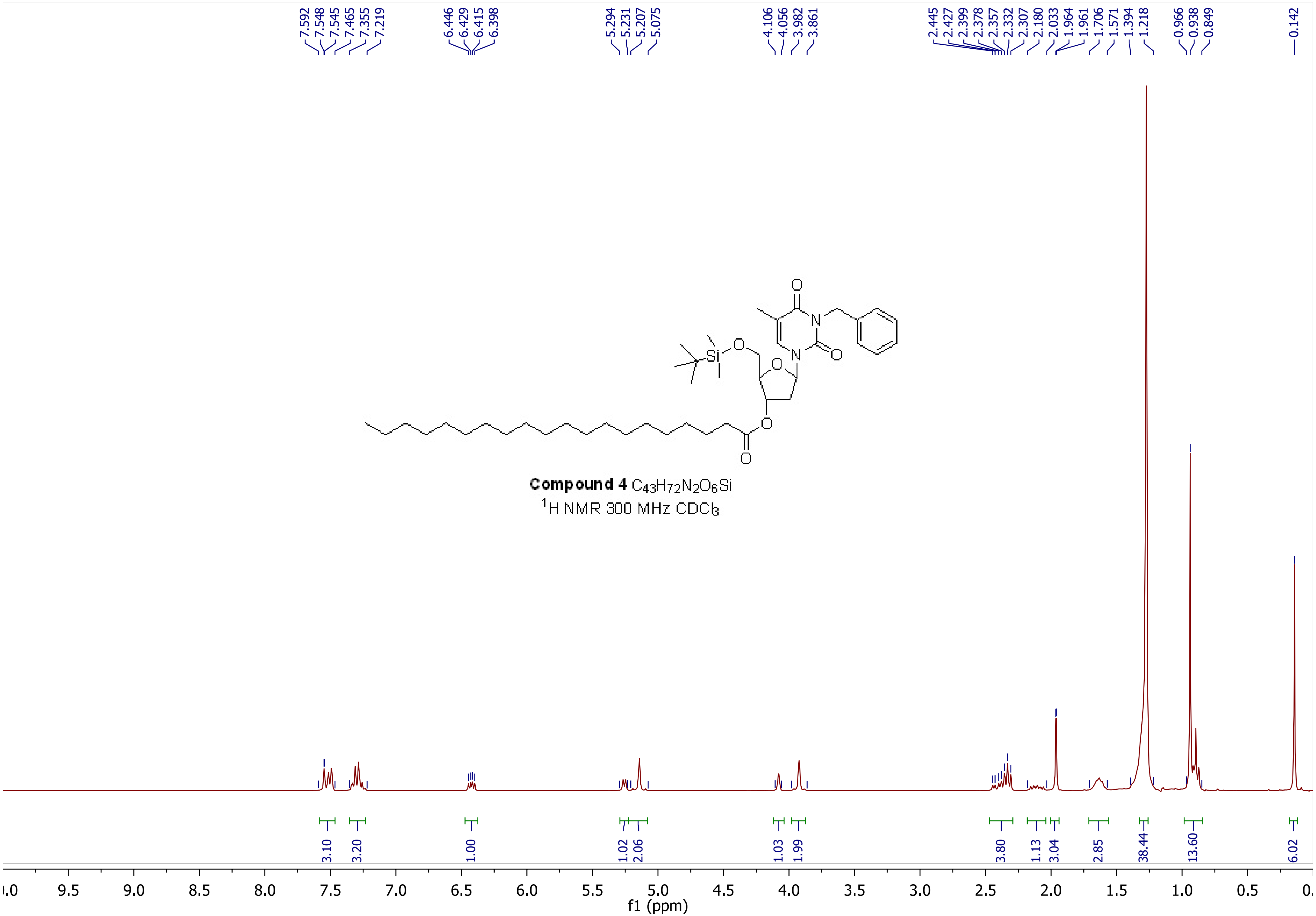
7.592 7.548 7.545 7.465 7.355 7.219
6.446 6.429 6.415 6.398
5.294 5.231 5.207 5.075
4.106 4.056 3.982 3.861
2.445 2.427 2.399 2.378 2.357 2.332 2.307 2.180 2.033 1.964 1.961 1.706 1.571 1.394 1.218
0.966 0.938 0.849
0.142
O
N
N
O
Si
O
O
O
O
Compound 4 $C_{43}H_{72}N_2O_6Si$
^{1}H NMR 300 MHz $CDCl_3$
3.10 3.20 1.00 1.02 2.06 1.03 1.99 3.80 1.13 3.04 2.85 38.44 13.60 6.02
9.5 9.0 8.5 8.0 7.5 7.0 6.5 6.0 5.5 5.0 4.5 4.0 3.5 3.0 2.5 2.0 1.5 1.0 0.5
f1 (ppm)

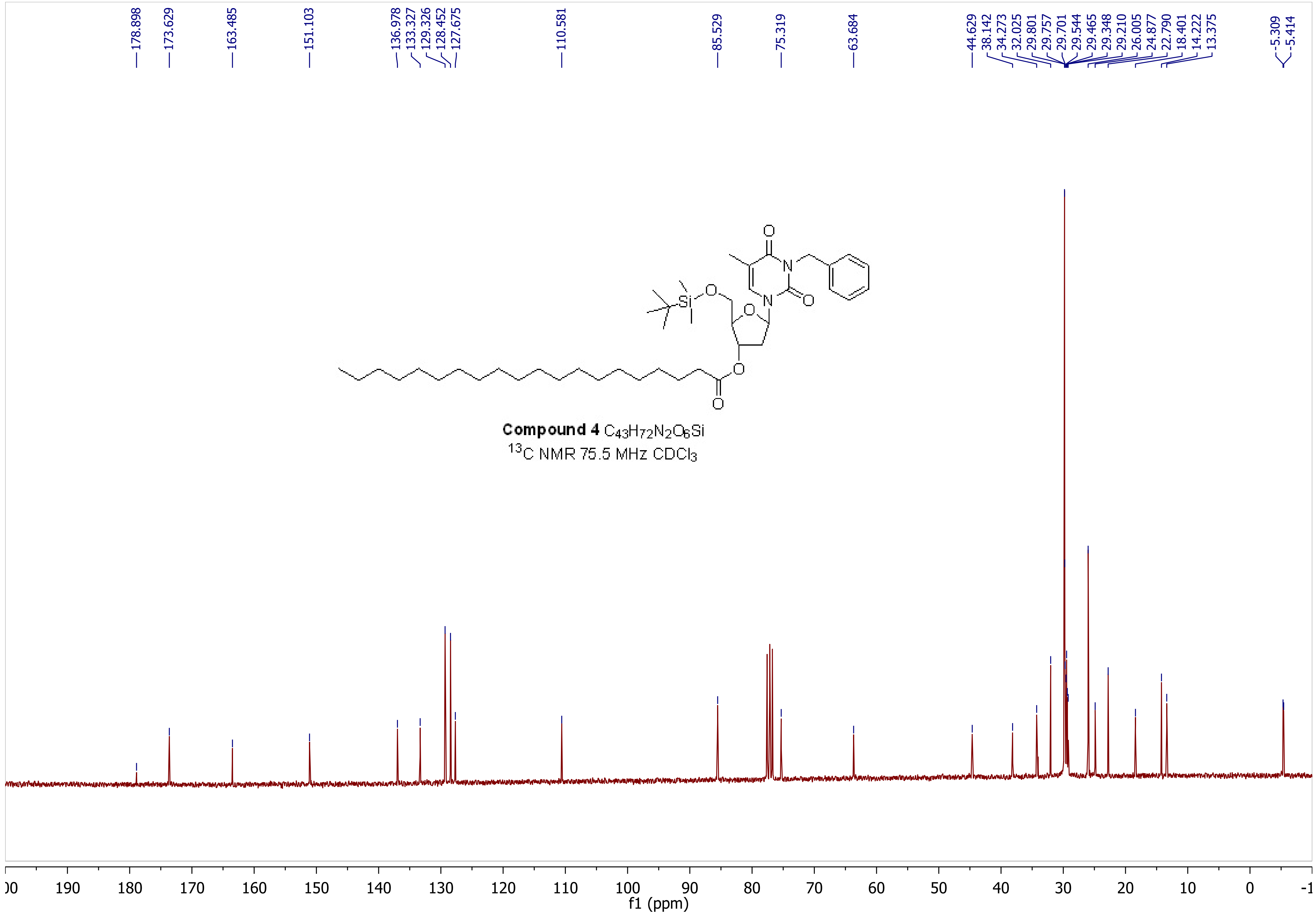
178.898
173.629
163.485
151.103
136.978
133.327
129.326
128.452
127.675
110.581
85.529
75.319
63.684
44.629
38.142
34.273
32.025
29.801
29.757
29.701
29.544
29.465
29.348
29.210
26.005
24.877
22.790
18.401
14.222
13.375
-5.309
-5.414
Compound 4 C43H72N2O6Si
13C NMR 75.5 MHz CDCl3
f1 (ppm)

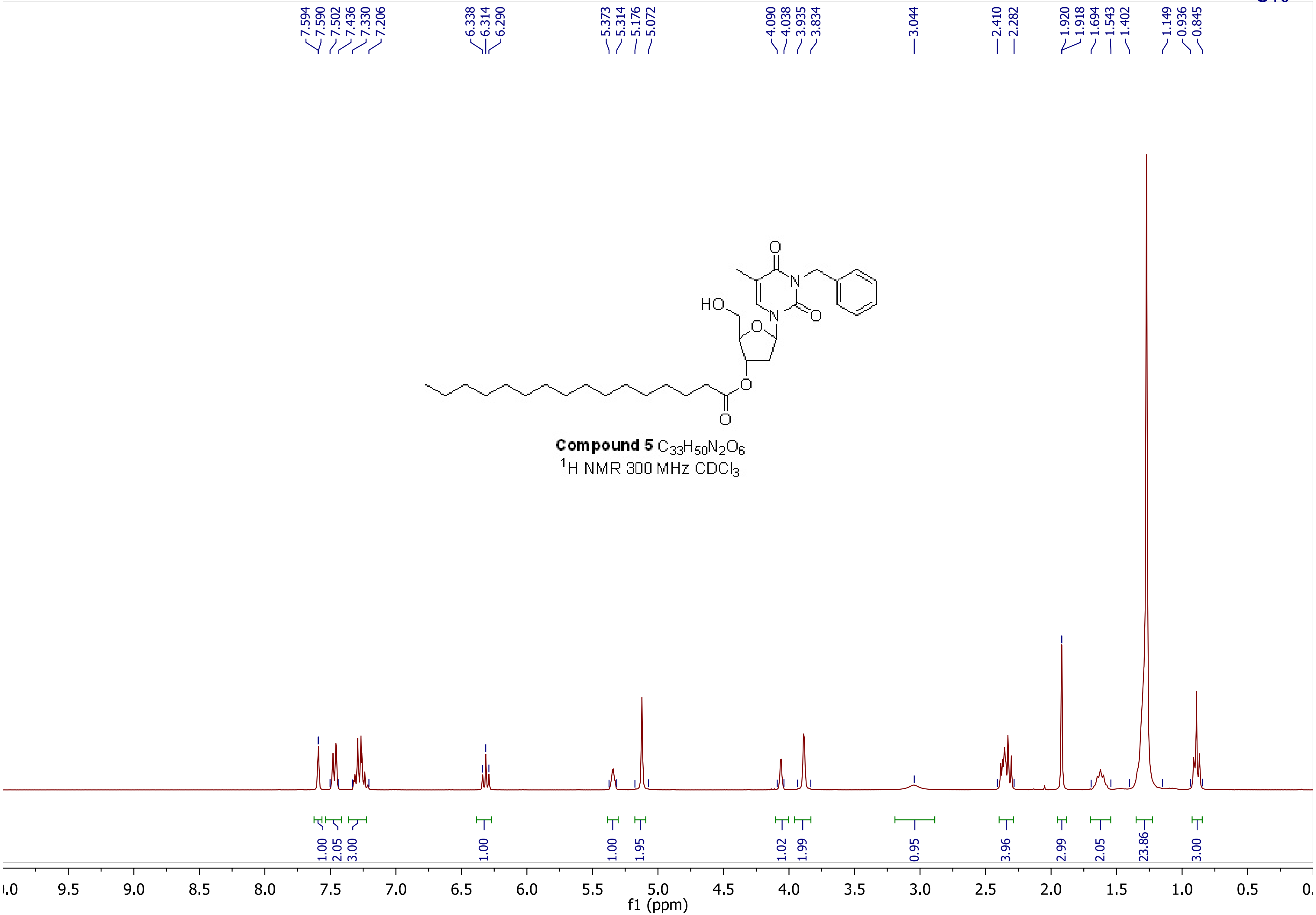
7.594 7.590 7.502 7.436 7.330 7.206
6.338 6.314 6.290
5.373 5.314 5.176 5.072
4.090 4.038 3.935 3.834
3.044
2.410 2.282
1.920 1.918 1.694 1.543 1.402
1.149 0.936 0.845
HO
O
N
Compound 5 C33H50N2O6
1H NMR 300 MHz CDCl3
1.00 2.05 3.00 1.00 1.00 1.95 1.02 1.99 0.95 3.96 2.99 2.05 23.86 3.00
9.5 9.0 8.5 8.0 7.5 7.0 6.5 6.0 5.5 5.0 4.5 4.0 3.5 3.0 2.5 2.0 1.5 1.0 0.5
f1 (ppm)

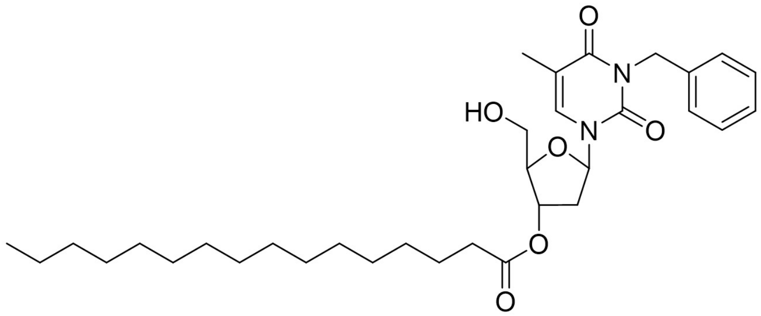

Compound 5 $C_{33}H_{50}N_2O_6$

^{13}C NMR 75.5 MHz $CDCl_3$

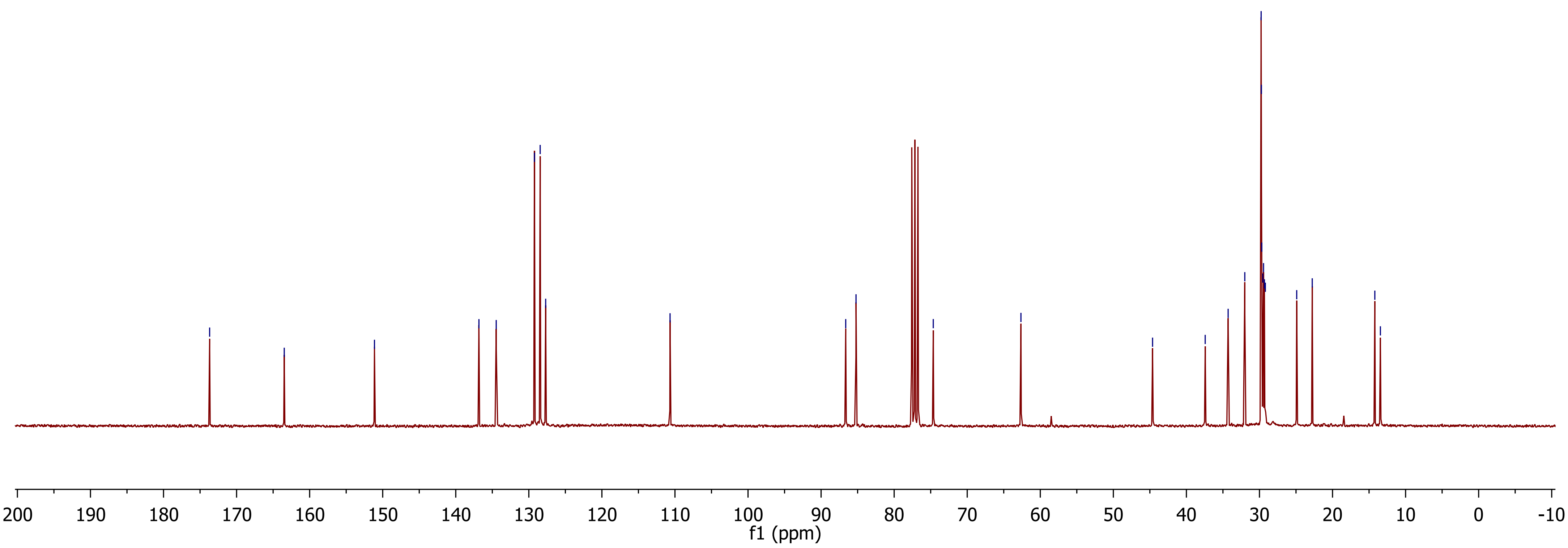

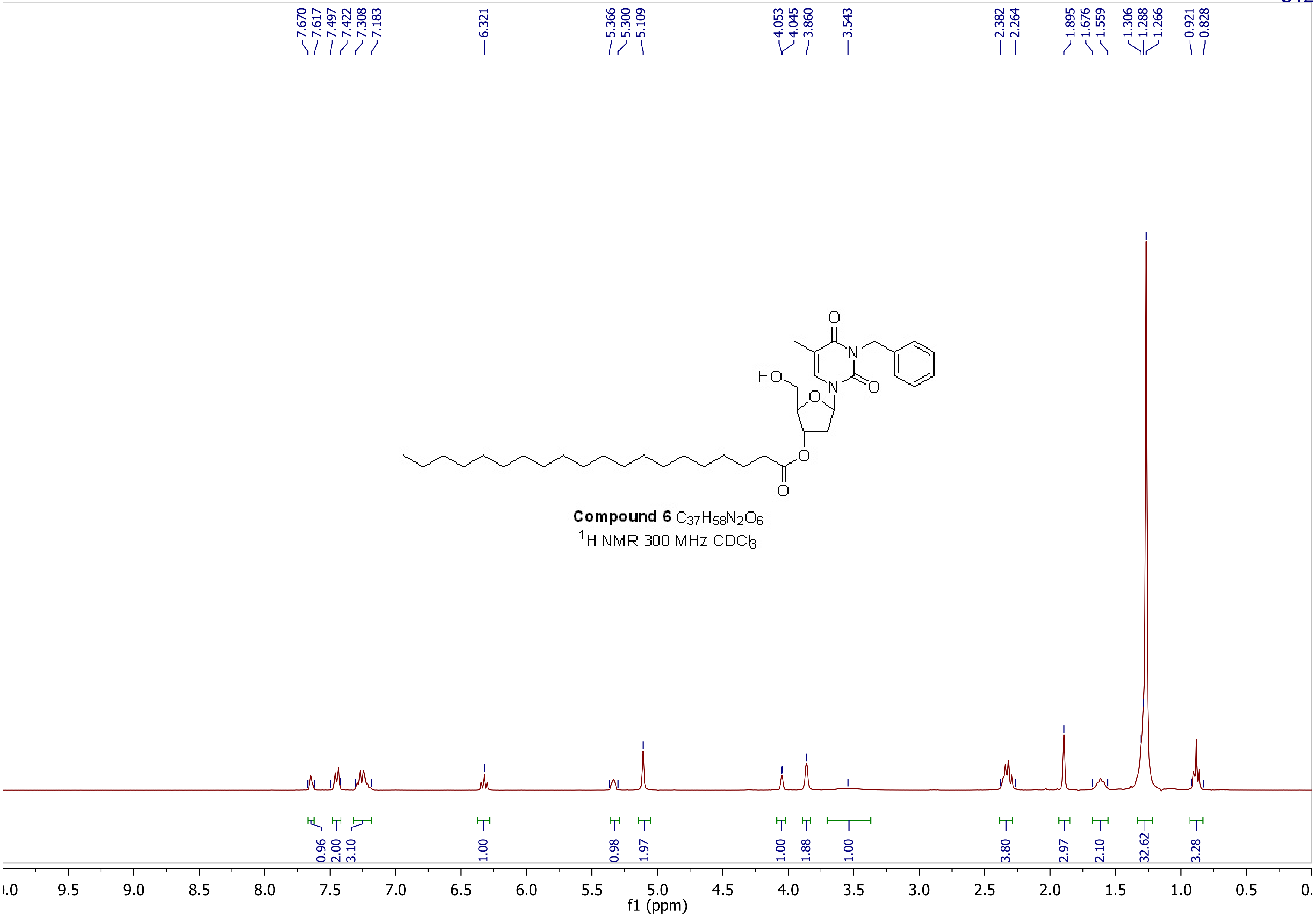

Compound 6 $C_{37}H_{58}N_2O_6$
^{1}H NMR 300 MHz $CDCl_3$

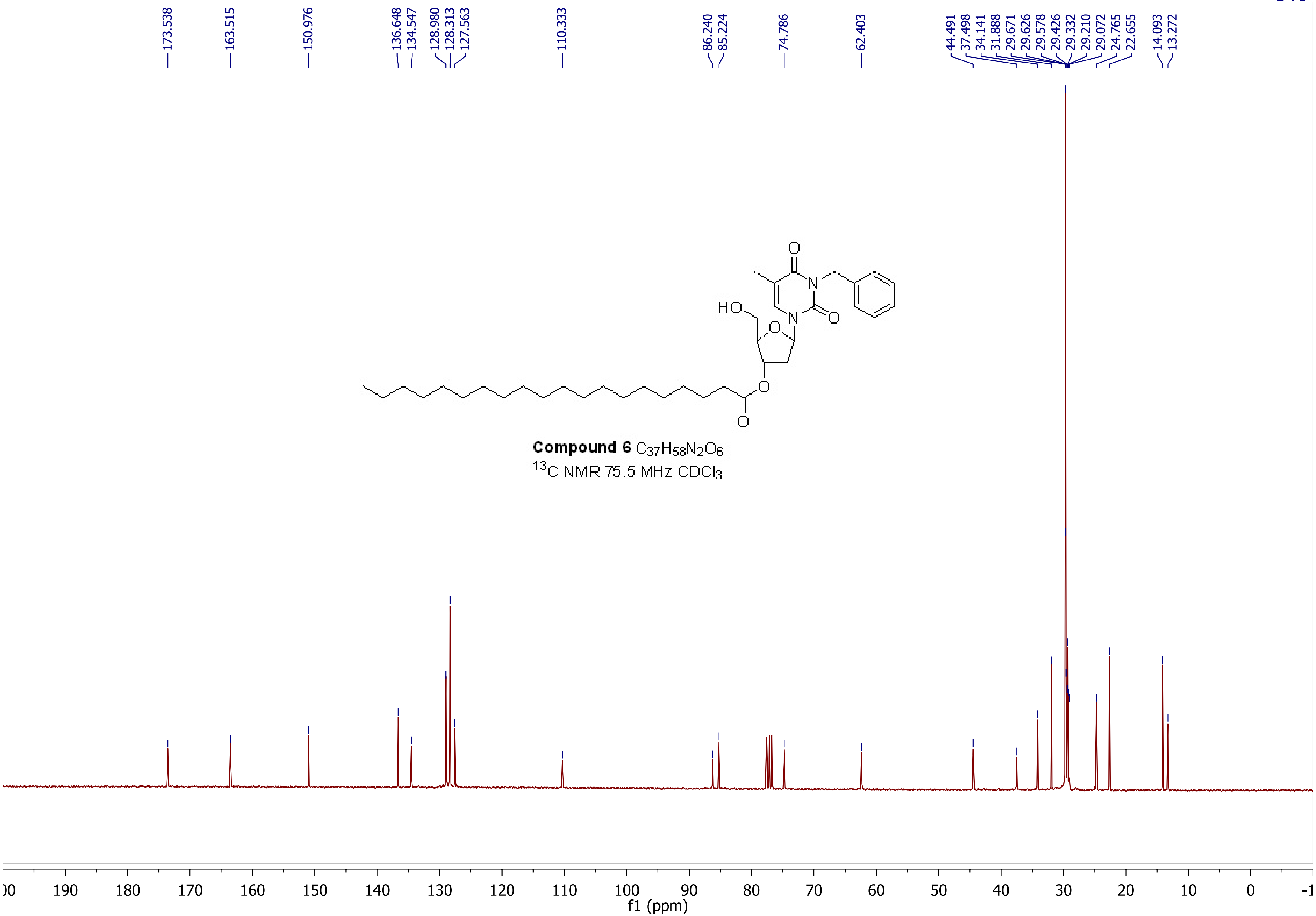
173.538
163.515
150.976
136.648
134.547
128.980
128.313
127.563
110.333
86.240
85.224
74.786
62.403
44.491
37.498
34.141
31.888
29.671
29.626
29.578
29.426
29.332
29.210
29.072
24.765
22.655
14.093
13.272
HO
O
N
O
O
O
O
Compound 6 C37H58N2O6
13C NMR 75.5 MHz CDCl3
f1 (ppm)
190
180
170
160
150
140
130
120
110
100
90
80
70
60
50
40
30
20
10
0
-1

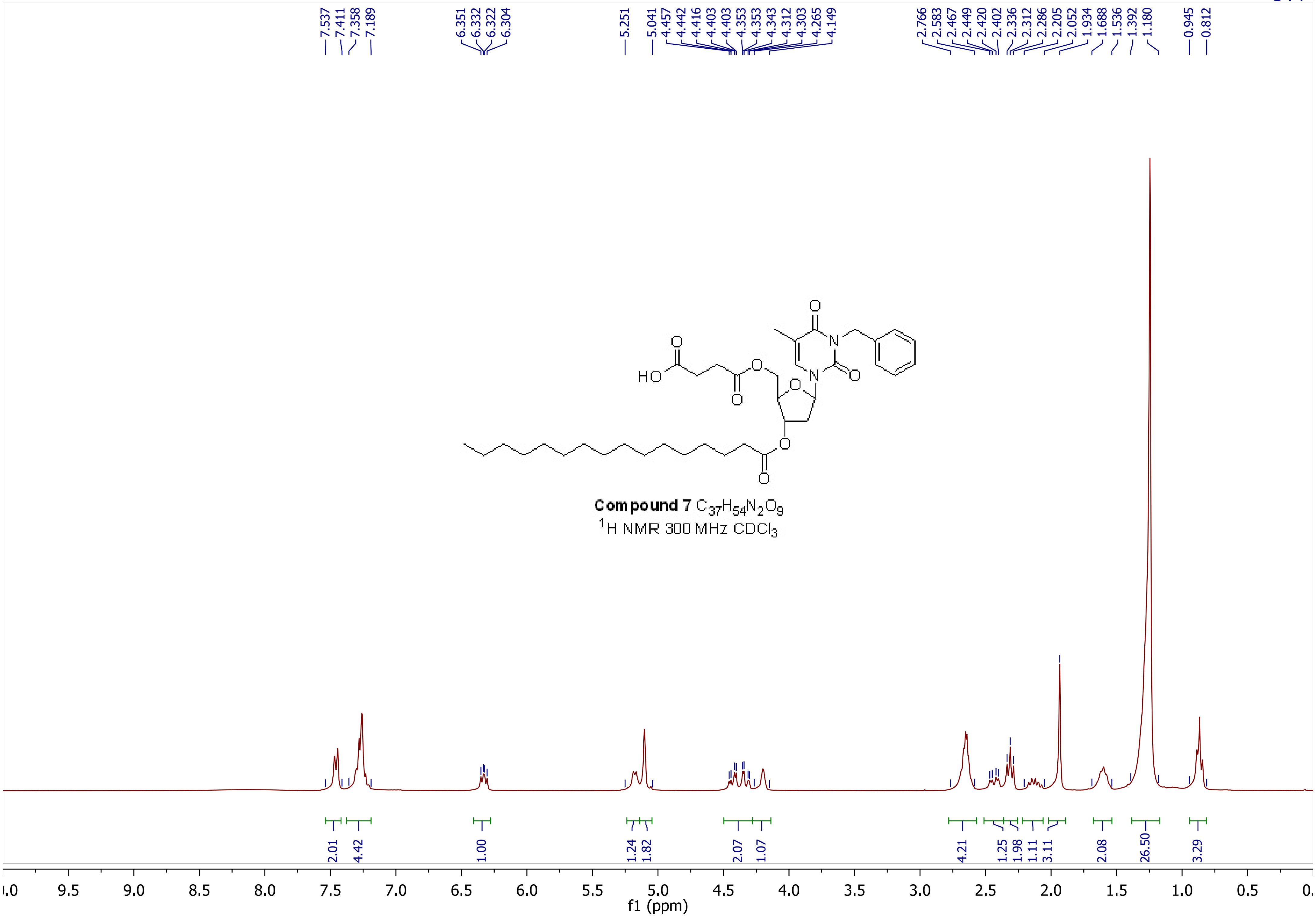
7.537
7.411
7.358
7.189
6.351
6.332
6.322
6.304
5.251
5.041
4.457
4.442
4.416
4.403
4.403
4.353
4.353
4.343
4.312
4.303
4.265
4.149
2.766
2.583
2.467
2.449
2.420
2.402
2.336
2.312
2.286
2.205
2.052
1.934
1.688
1.536
1.392
1.180
0.945
0.812
HO
Compound 7 $C_{37}H_{54}N_2O_9$
^1H NMR 300 MHz $CDCl_3$
2.01
4.42
1.00
1.24
1.82
2.07
1.07
4.21
1.25
1.98
1.11
3.11
2.08
26.50
3.29
9.5
9.0
8.5
8.0
7.5
7.0
6.5
6.0
5.5
5.0
4.5
4.0
3.5
3.0
2.5
2.0
1.5
1.0
0.5
f1 (ppm)

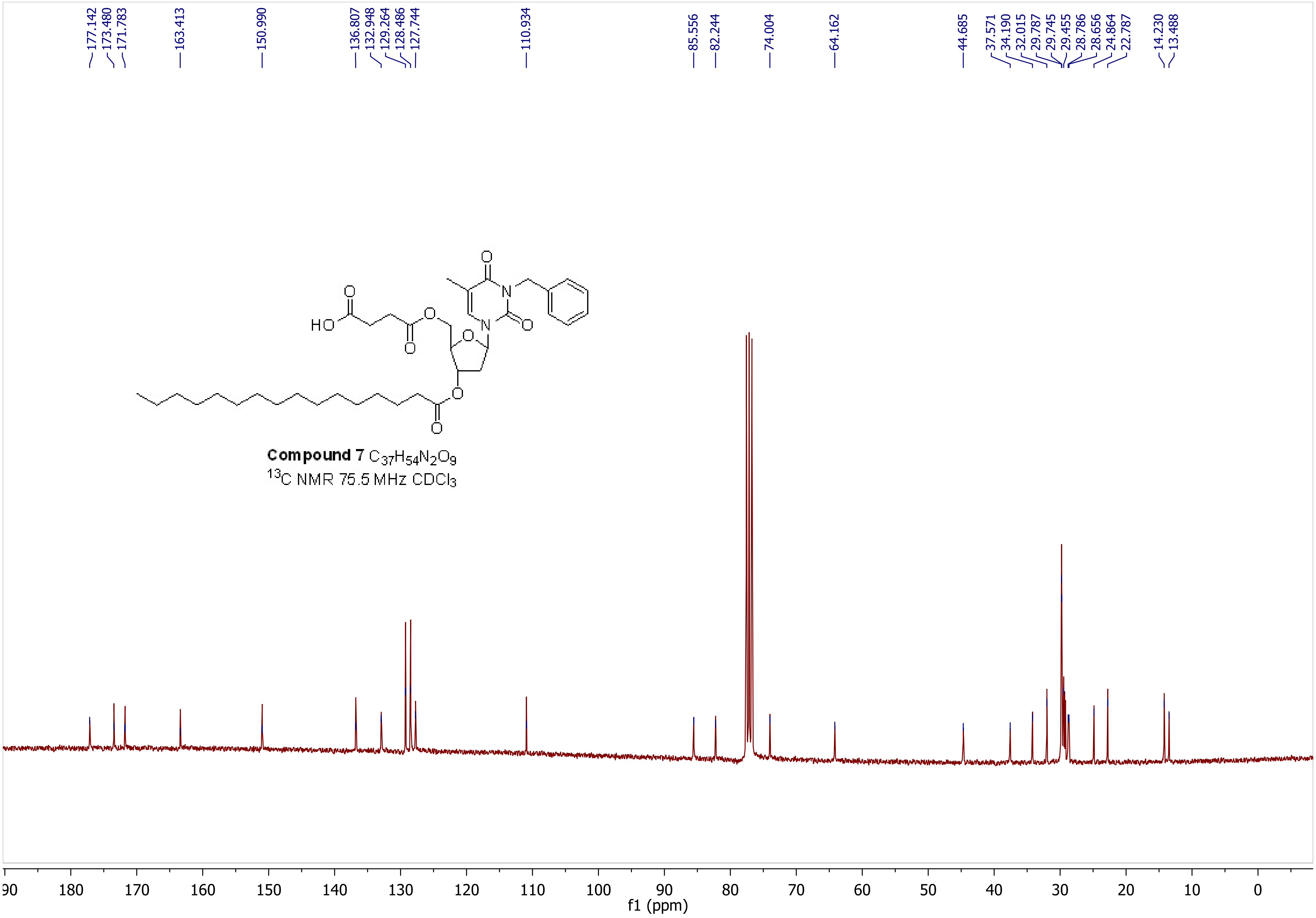

177.142
173.480
171.783
163.413
150.990
136.807
132.948
129.264
128.486
127.744
110.934
85.556
82.244
74.004
64.162
44.685
37.571
34.190
32.015
29.787
29.745
29.455
28.786
28.656
24.864
22.787
14.230
13.488
HO
O
N
Compound 7 C37H54N2O9
13C NMR 75.5 MHz CDCl3
f1 (ppm)

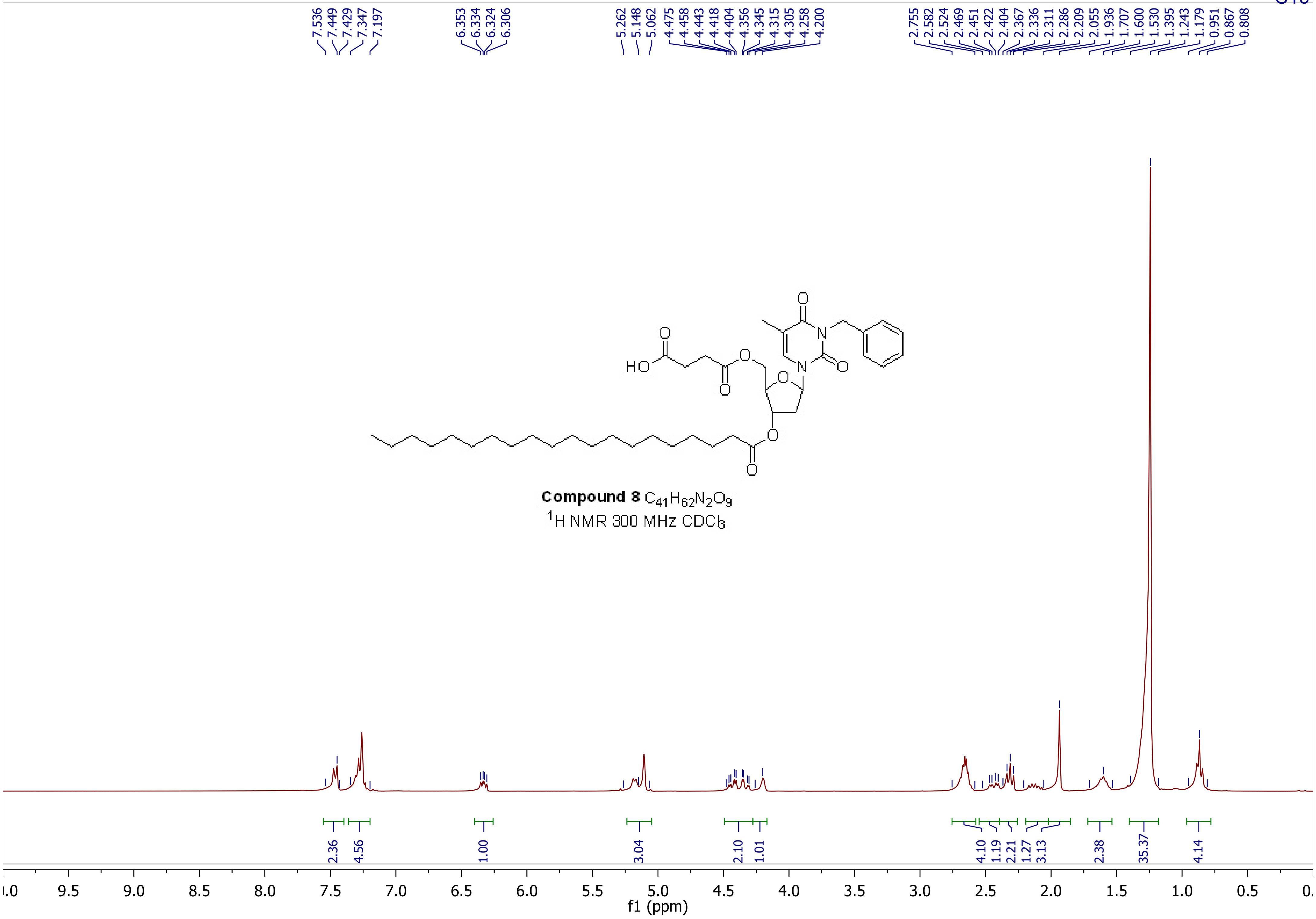
7.536
7.449
7.429
7.347
7.197
6.353
6.334
6.324
6.306
5.262
5.148
5.062
4.475
4.458
4.443
4.418
4.404
4.356
4.345
4.315
4.305
4.258
4.200
2.755
2.582
2.524
2.469
2.451
2.422
2.404
2.367
2.336
2.311
2.286
2.209
2.055
1.936
1.707
1.600
1.530
1.395
1.243
1.179
0.951
0.867
0.808
HO
O
N
Compound 8 C41H62N2O9
1H NMR 300 MHz CDCl3
2.36
4.56
1.00
3.04
2.10
1.01
4.10
1.19
2.21
1.27
3.13
2.38
35.37
4.14
9.5
9.0
8.5
8.0
7.5
7.0
6.5
6.0
5.5
5.0
4.5
4.0
3.5
3.0
2.5
2.0
1.5
1.0
0.5
f1 (ppm)

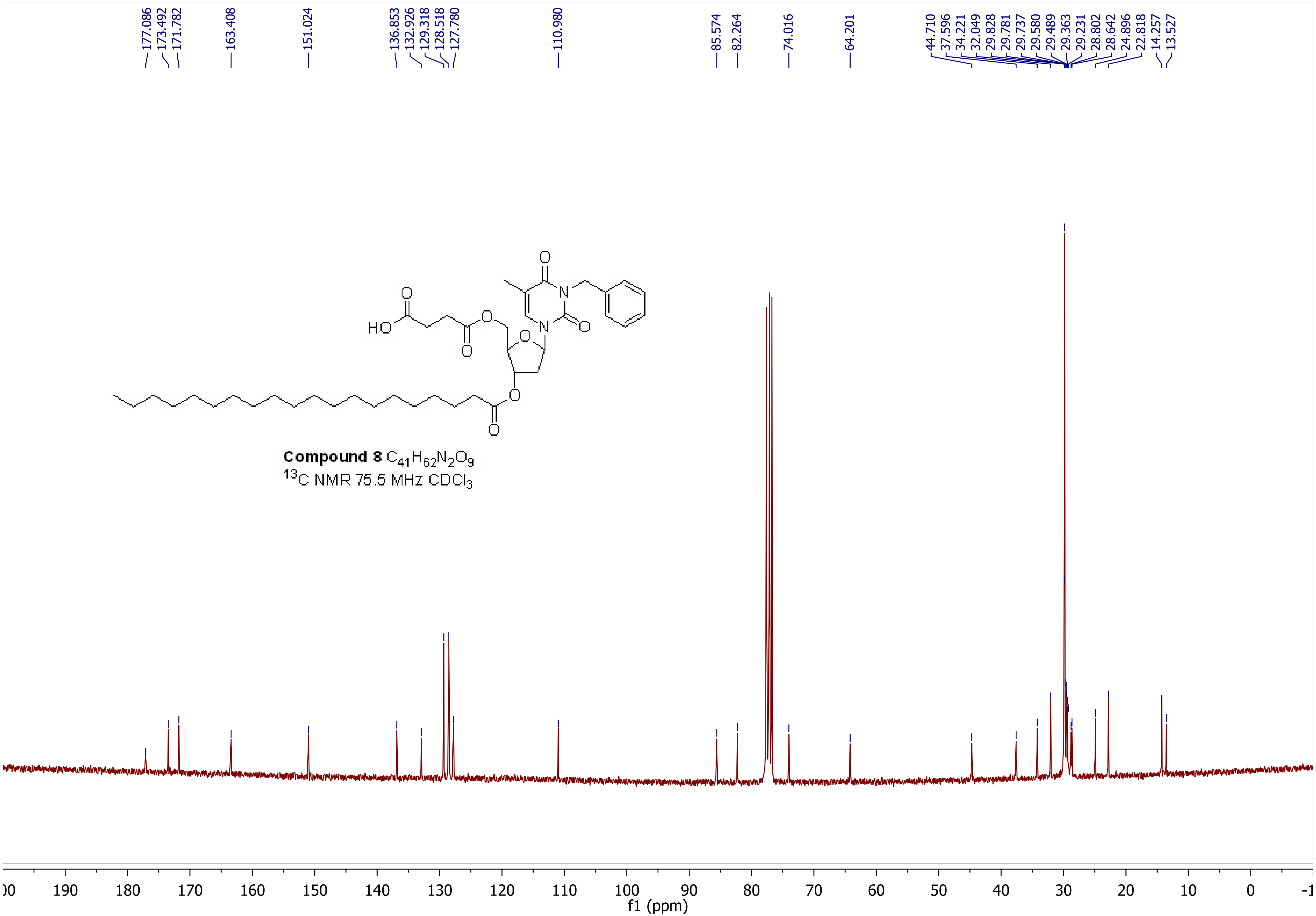
177.086
173.492
171.782
163.408
151.024
136.853
132.926
129.318
128.518
127.780
110.980
85.574
82.264
74.016
64.201
44.710
37.596
34.221
32.049
29.828
29.781
29.737
29.580
29.489
29.363
29.231
28.802
28.642
24.896
22.818
14.257
13.527
HO
O
N
Compound 8 $C_{41}H_{62}N_2O_9$
^{13}C NMR 75.5 MHz $CDCl_3$
f1 (ppm)
